# How are local authorities responding to climate change and health impacts? a policy document analysis of four England regions

**DOI:** 10.1186/s12889-026-28752-9

**Published:** 2026-07-31

**Authors:** Turner GA, O’Connor L, O’Connell E, Coleman PC, Sharma-Waddington H, Hajat S, Kovats RS

**Affiliations:** 1 NIHR Health Protection Research Unit in Climate Change and Health Security, 15-17 Tavistock Place, St Pancras, London, WC1H 9SH UK; 2 Department of Public Health, Environment and Society, London School of Hygiene and Tropical Medicine, London, UK; 3 Extreme Events and Health Protection, UK Health Security Agency, London, UK; 4 Department of Population Health, London School of Hygiene and Tropical Medicine, London, UK

**Keywords:** Climate change, Local government, Intervention, Public health, Policy, England

## Abstract

**Background:**

Climate change poses growing risks to population health. In England, local authorities can influence key determinants of climate-related health outcomes through responsibilities for housing, transport, environmental protection and public health. However, climate mitigation and adaptation are not statutory duties, and there is limited evidence on how climate and public health objectives are integrated across local policy frameworks.

**Aim:**

This study reviews local authority policy documents across English local governments, examining how they incorporate public health objectives within climate-related strategies, align policies across multiple policy domains and address health inequalities.

**Methods:**

We conducted a qualitative content analysis of 129 publicly available policy documents from 12 local authorities across four regions of England. Documents were identified through structured web searches and analysed using an a priori conceptual framework covering mitigation, adaptation, population health, environmental exposures and sectoral policy domains, vulnerability and inequality, and interventions.

**Results:**

Policy documents included a wide range of interventions, particularly in transport, housing energy efficiency and urban greenspace. Public health was rarely articulated as a primary objective and was more commonly framed as a co-benefit. Cross-sectoral alignment varied between policy domains, with stronger integration observed in active travel, housing retrofit, greenspace access and quality as well as flood resilience strategies. Air quality and transport strategies most clearly linked climate and health objectives, while food, greenspace and health and wellbeing strategies often did so implicitly. No authority had a standalone heat strategy, although heat-related actions appeared across multiple policy documents.

**Conclusion:**

Local authorities are implementing many interventions with potential public health benefits, but explicit integration of public health objectives within local climate policy frameworks remains variable. Stronger cross-sectoral coordination, clearer public health framing, and improved monitoring may help maximise health co-benefits and reduce climate-related health inequalities.

**Supplementary Information:**

The online version contains supplementary material available at https://doi.org/10.1186/s12889-026-28752-9.

## Background

Climate change is increasingly recognised as a major threat to population health and health systems worldwide. Climate-related hazards affect public health through multiple pathways, including the direct impacts of high temperatures and flooding, as well disruption to critical services, and health and social care services. Climate change will also affect air quality, food safety and security, and the transmission dynamics of infectious disease patterns. These impacts disproportionately affect populations already facing social, economic and environmental disadvantage, including older adults, low-income households, people with chronic health conditions and communities exposed to poorer environmental conditions. In the United Kingdom, rising temperatures, more frequent heatwaves, more intense rainfall events, and flooding are the most significant climate-related hazards affecting population health [1]. Without sufficient adaptation, projections suggest that heat-related mortality in England and Wales could increase substantially over the coming decades, particularly among older adults and people with pre-existing health conditions [2, 3].

Local authorities play a central role in the implementation of climate adaptation and mitigation as many climate policies fall within their legal remit, for example through spatial planning and housing. Sectoral responsibilities for housing, transport, food systems, environmental protection and the management of green and open spaces shape key determinants of health including air quality, physical activity, access to healthy food and exposure to environmental hazards. Since 2013, local authorities in England have also held primary responsibility for many public health functions [4]. Public health teams now work alongside planning, housing and environmental departments and collaborate with health and social care delivery partners through Integrated Care Systems. These arrangements position local government as a key actor in delivering both emissions reduction and adaptation measures to protect and promote the health of their communities [5].

Despite their important role in implementation, climate mitigation and adaptation are not currently explicit statutory duties for local authorities in England, although many existing statutory responsibilities implicitly require consideration of climate-related risks and resilience. National policy frameworks such as the UK National Adaptation Programme does not impose legal requirements on local or national government to meet adaptation targets or implement specific measures. In contrast, the UK has national legally binding national emissions reduction targets. At the local level, councils hold statutory responsibilities relevant to climate resilience, including spatial planning, housing standards and building regulation, environmental protection and emergency preparedness [5]. All these responsibilities are necessary for effective adaptation to reduce the impact of climate change on human health but are not necessarily labelled as adaptation actions. Adaptation and mitigation need to be considered at the same time. Some mitigation measures can undermine adaptation, particularly housing retrofit and energy efficiency improvements, which increase the risk of overheating and poor indoor air quality [6, 7].

Much of the momentum in local climate and health action has emerged through voluntary initiatives, community activism, and local political leadership. A key driver has been the rapid spread of climate emergency declarations by local authorities. By 2023 more than 300 local authorities had adopted such declarations [8], which often include emissions reductions target for the local authority, and, more rarely, for the population as whole. The declarations do not confer additional statutory powers or funding [9, 10] and in some cases local authorities have rescinded these declarations [11]. The ability of local authorities to deliver local climate interventions is shaped by wider structural constraints. Over the past decade, local government funding in England has declined substantially in real terms, resulting in reduced staffing capacity and increasing pressure on statutory services such as adult social care, housing, and education [12–14], while ring fenced public health funding is allocated for delivery of core public health duties (health protection, sexual health services). Furthermore, local areas are often well prepared for hazards they experience regularly, but less prepared for emerging or infrequent risks [15, 16].

Research highlights substantial variation in institutional resources and capabilities between UK local authorities in the delivery of net zero [17]. Climate initiatives are therefore often delivered through short-term project funding or external grants rather than sustained core budgets [8]. The Third UK Climate Change Risk Assessment highlighted that adaptation progress is constrained by limited local governance structures relative to the scale of climate risks facing UK communities [8, 18].

Local climate planning often lacks a robust evidence base on health impacts and inequalities, and there remains limited understanding of how climate and public health objectives are integrated across local policy frameworks [19]. Surveys also indicate that air pollution and severe flooding are among the climate-related health risks that communities most expect local governments to address [20]. Translating national emissions commitments into coordinated local action that maximises health benefits such as improved air quality, increased physical activity, reduced exposure to excess heat and improved thermal comfort remains a key challenge.

Effective local responses require cross-sector collaboration, strong strategic leadership and improved public health intelligence to assess vulnerability, target interventions and monitor outcomes. Local authority strategies and policy documents provide an important window into how climate risks and responses are conceptualised within governance systems. These documents articulate priorities, responsibilities and planned interventions across sectors such as planning, housing, transport and public health, and therefore reveal how climate and health objectives are integrated within formal policy frameworks. Previous research has highlighted considerable variation in how local authorities recognise and address the health impacts of climate change within adaptation planning [5], and recent analyses suggest that many local climate action plans continue to treat health primarily as a co-benefit to mitigation rather than a central policy objective [9]. At the same time, local governments report significant gaps in the evidence base needed to support climate and public health decision-making and prioritisation of interventions [19]. This study contributes descriptive evidence on how local authorities in England frame public health priorities within climate actions across policy documents, and examines how objectives and interventions are aligned across sectors relevant to population health and addressing health inequalities.

## Methods

### Study design and setting

We undertook a content analysis of local authority policy documents in England to examine both proactive mitigation and adaptation planning, as well as preparedness for climate-related risks. We particularly focused on the identification of interventions and the alignment of objectives across sectors. Document content analysis is a systematic method for reviewing and interpreting printed and electronic materials, and is widely used in public health and policy research to assess governance approaches and implementation priorities [21]. This study builds on established approaches used to analyse local government climate strategies, adaptation plans, and public health policies, and draws conceptually on frameworks linking climate hazards, exposure pathways, vulnerability, and policy responses [22–24].

This study focused on local authorities in England which has a complex local government system with several layers depending on the region, with public health responsibilities sitting in different levels depending on the type of local authority (Table 1). Four regions were purposively selected to capture geographic, demographic, and governance diversity: London, the South East, the North West, and the North East of England. Within each region, a minimum of three local authorities were included. Selection aimed to ensure variation in: local authority type (e.g. unitary, metropolitan, upper-tier county), urban or rural context and socio-economic profile, while maintaining feasibility for in-depth document review.

**Table 1:** Government structure of local authorities in England

Type of Local Authority	Jurisdiction	Responsibilities	Public Health Role
County Council (upper-tier council)	Non-metropolitan counties	Education, transport, highways, social services, libraries, waste disposal, trading standards, strategic planning	Public health services (e.g., health promotion, sexual health, vaccinations, obesity programs)
District / Borough / City Council (lower-tier council)	Non-metropolitan counties (within counties)	Housing, local planning, waste collection & recycling, environmental health, leisure services, council tax collection	No
Unitary Authority (single-tier council)	Large towns/cities & some counties	Combines county and district responsibilities: education, transport, housing, planning, social services, waste management	All local public health functions
Metropolitan Borough Council (single-tier council)	Large urban/metropolitan areas (e.g., Greater Manchester, West Midlands)	All local services for the borough	Public health within the borough
London Borough Council (single-tier council)	Greater London (32 boroughs)	Education, social services, housing, local planning, waste management, leisure	Borough-level public health (strategic oversight from London Health Board/GLA)
Parish / Town Council (lowest-tier council)	Rural areas and smaller towns	Local amenities (parks, community centres), consultation on planning, footpaths, street lighting, community events	No
Combined Authority (collaborative across multiple authorities)	Optional, often in metropolitan areas	Strategic transport, economic development, skills, regeneration	No (may influence health through regional strategies)

### Search strategy and inclusion criteria

We conducted a structured search for publicly available policy and strategy documents produced by each selected local authority. The document types included: climate change or climate action strategies; climate adaptation or resilience plans; net zero or carbon neutrality strategies; public health strategies and Joint Strategic Needs Assessments (JSNAs) and sector-specific strategies relevant to climate and health (e.g. transport, housing, food, green infrastructure). To identify these documents, keyword searches were conducted using Google and local authority website search functions using combinations of the terms “climate change”, “health”, “adaptation”, “net zero”, “resilience”, and sectoral domains relevant to climate mitigation and health (including food systems, housing, and transport). Researchers hand-searched local authority webpages for additional strategies, plans and policies which may not have been included in the web search. Documents were included if they: were current or recently superseded at the time of review and contained explicit objectives, policies, or actions relating to climate change mitigation or adaptation. The review focused on policy domains with direct relevance to either climate mitigation or adaptation and population health pathways, including housing, transport, food systems, greenspace, air quality and public health. Broader local authority functions such as emergency planning, adult social care and general waste management were not systematically reviewed because they were less consistently represented in standalone policy documents across authorities and would substantially broaden the scope of analysis.

In this paper, the term “policy documents” is used as an umbrella term encompassing strategies, plans, frameworks and related governance documents produced by local authorities. Where relevant, specific document types (e.g. local plans, transport strategies, adaptation plans or Joint Strategic Needs Assessments) are referred to individually. The documents included in this review are the most recent version as of December 2025. Some local authorities published multiple policy documents for each sector as well as local or corporate plans. Local plans are statutory spatial planning documents prepared by local planning authorities which guide land use, housing, infrastructure and development decisions within a local area. They are particularly relevant to climate mitigation, adaptation and public health because they shape the built environment and long-term patterns of environmental exposure. Planning was examined as a cross-sectoral function influencing multiple policy documents such as housing, transport, greenspace and flood risk management. Corporate plans are strategic documents published by local councils that set out their vision, priorities, objectives, and intended outcomes for guiding decision-making, service delivery, and resource allocation over a multi-year period.

### Conceptual framework

**Fig. 1:**
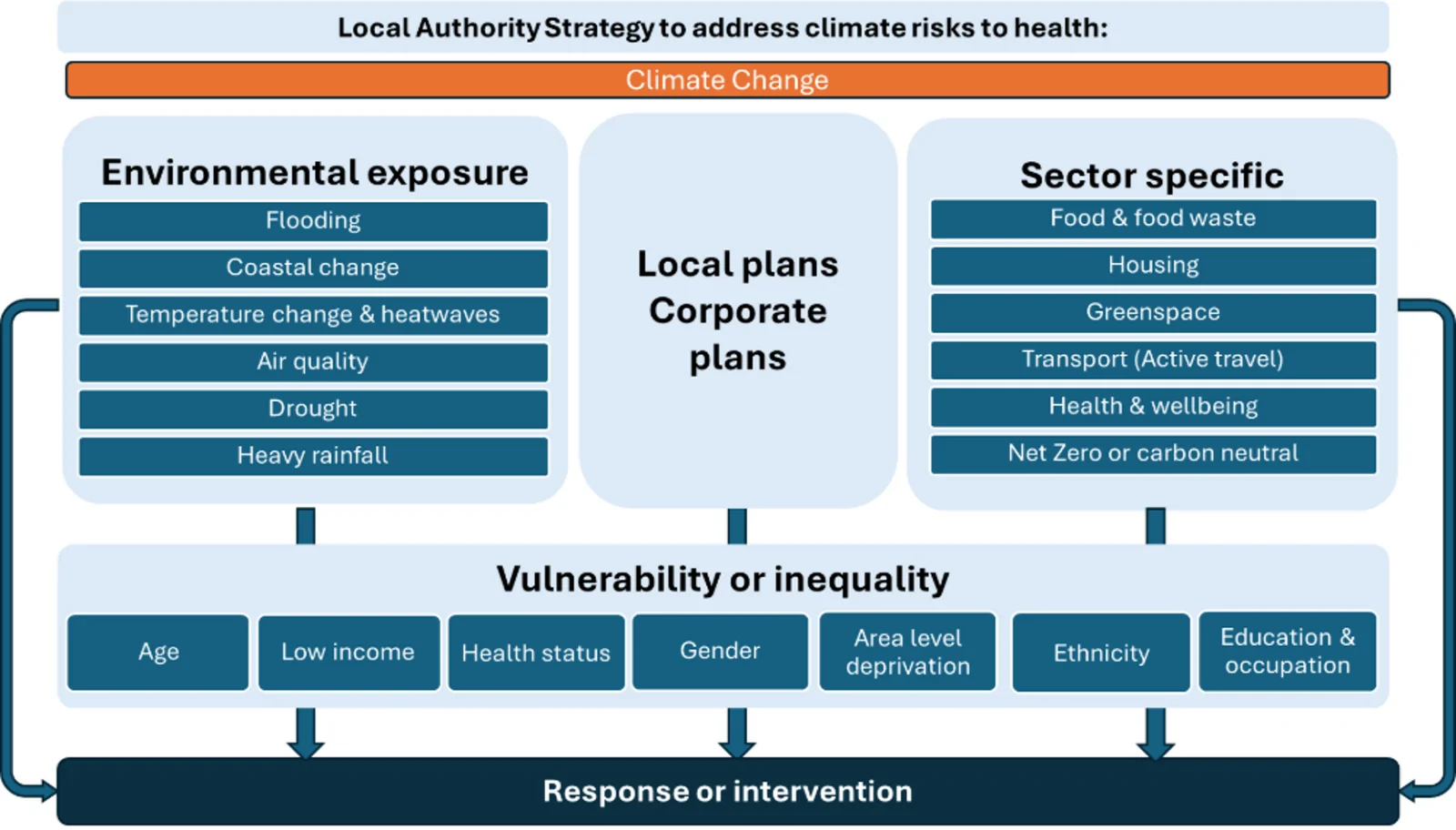
Conceptual matrix used to review interventions that address climate change, health and equity

Systematic document identification was guided by an a priori conceptual framework (Fig. 1) informed by existing climate–health models, including the UK Climate Change Risk Assessment [18] and the Health in all Policies framework [23]. The framework conceptualises local authority responses as operating across three interconnected domains:

1. Environmental exposure or hazard (e.g. heatwaves, flooding, air pollution);
2. Sectoral policy domains (e.g. housing, transport, food, greenspace, health and wellbeing);
3. Local and corporate level policy.
4. Vulnerability and inequality (e.g. older age, low income, health status, area-level deprivation including neighbourhood disadvantage beyond income).

Environmental exposure categories included both acute and chronic climate-related hazards relevant to population health in England, including flooding, coastal change, heat, air pollution and heavy rainfall. Wildfire was acknowledged as an emerging climate-related risk in England but was infrequently referenced within reviewed policy documents. The sectoral domains included in this framework were selected because they represent key areas through which local authorities influence both greenhouse gas emissions, climate resilience and determinants of population health. Policies relating to housing, transport, food systems, and greenspace shape factors such as air quality, physical activity, thermal comfort, and access to healthy environments, and therefore represent important pathways for delivering climate mitigation, adaptation, and public health co-benefits. Planning was treated as a cross-sectoral function influencing multiple domains including housing, transport, greenspace, flooding resilience and urban development, rather than as a standalone policy category.

### Data extraction and analysis

All included documents were reviewed and read in full by one researcher. Relevant policies, objectives, and actions were extracted and entered into a structured data extraction table in Excel. Each strategy underwent data extraction and binary coding of public health inclusion in intervention (1 = yes, 0 = no) according to pre-defined categories (Table 2). To assess cross-sectoral alignment, we examined the extent to which climate-health objectives and interventions were reflected consistently across policy domains and referenced across multiple policy documents within the same local authority. This included consideration of whether interventions were framed similarly across sectors, supported by implementation mechanisms, such as delivery plans, budget allocation, governance structures, or partnerships. This analysis focused on identifying synergies, gaps, and potential conflicts between mitigation and adaptation objectives, and between climate and public health priorities.

**Table 2:** Local authority strategy coding categories and description

Category	Description
Descriptive characteristics	Title, publication year, local authority type, region, document link
Strategy category	(1) Climate-change focused policy documents: climate action plans, adaptation strategies, resilience plans and net zero strategies (2) Sector-specific policy documents: housing, transport, air quality, greenspace, flooding, health and wellbeing strategies and food strategies (3) Local and corporate plans
Primary sector of strategy	Climate & environment, housing, transport, food and food waste, greenspace, health and wellbeing, local, Net zero/sustainability, air quality
Climate change & health focus	Direct references explicitly linked climate action to health outcomes, indirect references addressed determinants of climate-related health outcomes without an explicit health framing, and no reference indicated no substantive discussion of climate–health relationships.
Strategy alignment	Informed by other national or local policy documents and strategies
Targets and monitoring	Mitigation or adaptation targets, monitoring and evaluation of strategy
Health impacts and/or outcomes addressed in sectoral strategy	Mortality, morbidity; health service disruption
Intervention description	Policy commitment, programme delivery, investment in programmes, personnel and partnerships, workforce development, guidance and community-based initiatives
Population groups referenced	e.g. older people, low-income households, people with long-term conditions

Documents were classified as having a “direct” climate-health focus when they explicitly linked climate change or climate policies to local health outcomes or identified health as a stated policy objective. “Indirect” references referred to policies addressing determinants of climate-related health outcomes, such as housing quality, transport or greenspace, without explicitly framing these as health interventions. “No reference” indicated no substantive discussion of climate-health relationships.

## Results

### Overview of policy documents

We identified 129 policy and strategy documents across 12 local authorities in four regions of England. The number and type of strategies varied substantially between local authorities, with no authority having a complete set of strategies across all sectors relevant to climate and health. The most commonly identified strategies were flood risk management strategies (*n* = 15), health and wellbeing strategies (*n* = 15), local or corporate plans (*n* = 14), climate and environment strategies (*n* = 14), housing strategies (*n* = 14), transport or active travel strategies (*n* = 14), food and food waste plans (*n* = 13), air quality action plans (*n* = 11). Most local authorities had published a strategy related to greenspace, parks or open spaces (*n* = 13). Six strategies specifically outlined an action plan towards a low carbon or net zero local authority.

The reviewed policy documents varied considerably in purpose and scope. For the purposes of analysis, documents were grouped into three broad categories: (1) climate change-focused documents, including climate action plans, adaptation strategies, resilience plans and net zero strategies; (2) sector-specific policy documents, including housing, transport, air quality, greenspace, flooding, health and wellbeing strategies and food strategies; and (3) local plans and corporate plans. Climate-focused documents typically established overarching priorities and lacked health-specific targets, while sector-specific and local plans contained more detailed policies and implementation actions relevant to climate-related health outcomes.

### Integration of climate and public health objectives

**Fig. 2:**
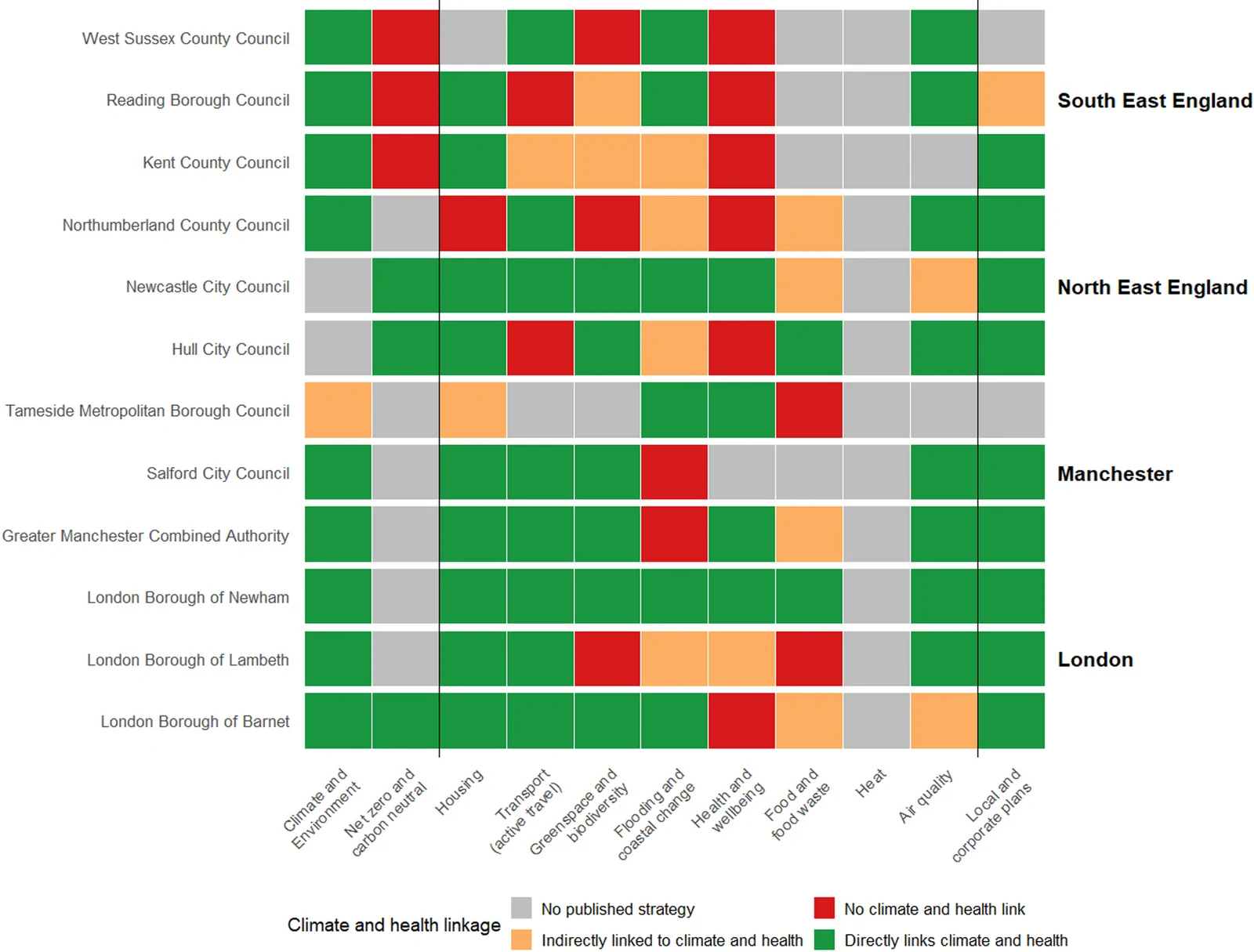
Local authority policy documents reviewed in how they link to health and climate change in the four regions in England by policy document type

Figure 2 shows how strategy documents referenced climate change and population health. Across the documents reviewed: 56% directly linked climate change and population health, 22% indirectly linked climate change to health outcomes and 22% made no reference to climate change and health. Public health was rarely articulated as a primary objective of climate action and was more commonly framed as a co-benefit across sector-specific policy documents including housing, transport, green infrastructure, air quality and food systems. Few policy documents included measurable health outcomes or indicators, and health impacts were rarely evaluated or monitored. In most local authorities, local plans, housing strategies and climate strategies explicitly linked climate risks to health outcomes including heat, flooding, air quality and fuel poverty.

Climate-change focused policy documents did not typically introduce new policies but collated actions from existing sector-specific strategies. Net zero strategies generally framed public health as a co-benefit of mitigation and adaptation through fuel poverty reduction, improved air quality and housing retrofit. In the sector-specific strategies, the air quality plans and active travel strategies were the most likely to make explicit connections between climate change and health (local emissions reduction and increased physical activity have major benefits to public health). In greenspace and food strategies, health benefits were often implicit. 50% of health and wellbeing strategies made no explicit links between climate change and health.

Differences were also observed between policy documents produced at regional and local governance levels. Policy documents developed by combined authorities (e.g. Greater Manchester) tended to frame climate and public health relationships at a strategic system level, particularly through transport decarbonisation, air quality improvement, and economic transition policies. In contrast, policy documents produced by constituent local authorities more frequently addressed climate-related health risks through operational determinants of health, including housing quality, fuel poverty, flooding, and access to greenspace. As a result, population health considerations were often more explicitly embedded within local authority sector-specific strategies, whereas regional strategies more commonly framed health as a co-benefit of wider climate objectives.

### Climate-health interventions across policy domains

**Fig. 3:**
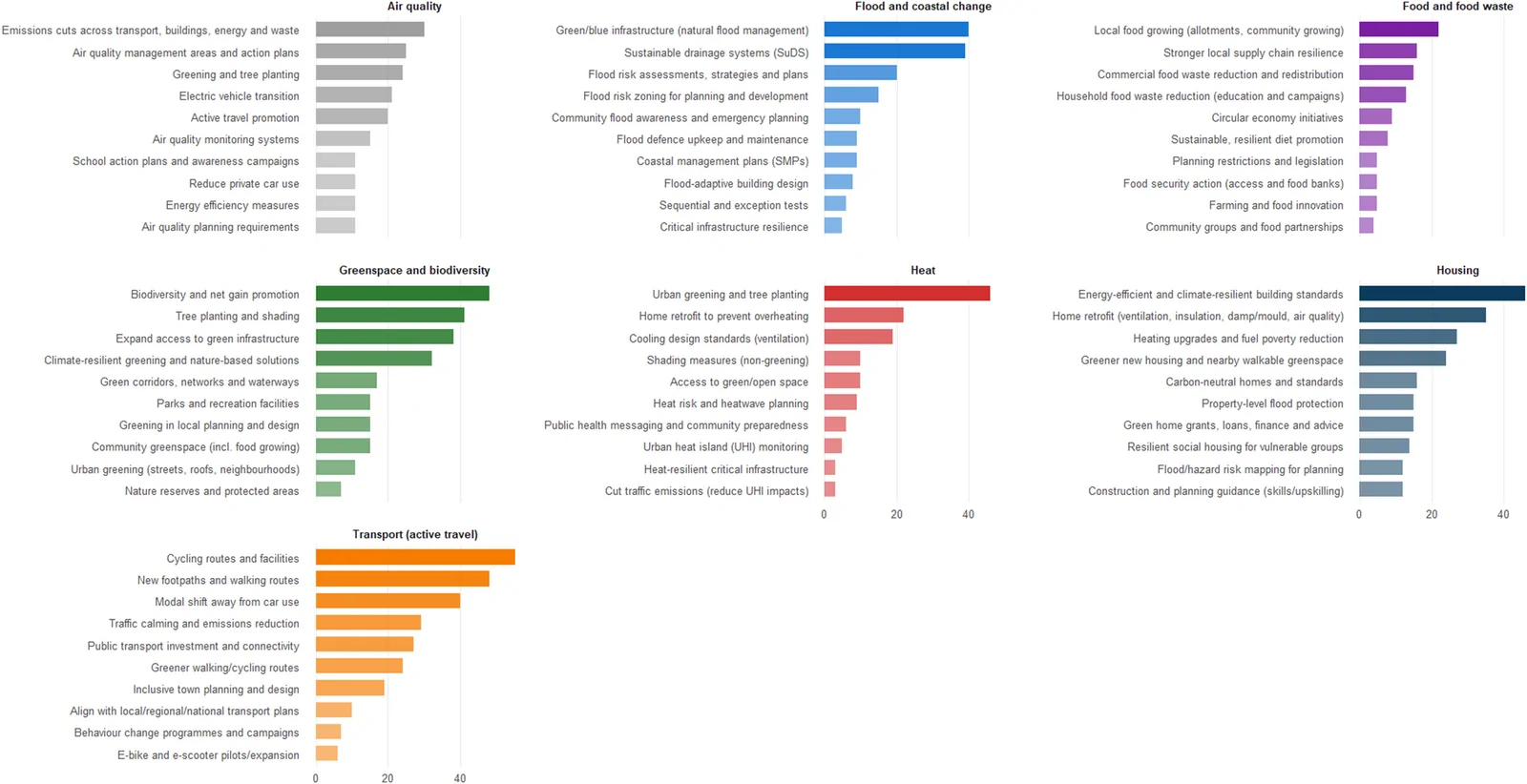
Top 10 types of climate change and public health related interventions identified across policy documents grouped by theme (full intervention list in supplementary material S1)

The review identified a wide range of interventions (Fig. 3), with the most frequently identified concentrated in transport, housing and greenspace (that is, actions promoting active travel, improving building energy efficiency and expanding urban greening). Interventions explicitly addressing heat risk (other than urban cooling through greening), food systems or health system preparedness were substantially less common. Housing intervention types included: energy efficiency retrofit measures (e.g., insulation, glazing, fabric-first upgrades, EPC improvement programmes), low carbon heating transitions (heat pumps, district heating, solar thermal, hydrogen-ready systems), and climate-resilient design approaches (overheating mitigation, flood resilient construction, SuDS integration, raised utilities, climate risk assessments), alongside regulatory enforcement and green infrastructure integration. Flooding and coastal change strategies were similarly multi-layered, comprising Sustainable Drainage System (SuDS) approaches, natural flood management measures, spatial planning controls (e.g. sequential tests, flood risk assessments, flood zone restrictions), and additional infrastructure, property-level, monitoring, and emergency preparedness measures. Air quality interventions were dominated by transport emission reduction measures such as clean air zones, complemented by monitoring systems and regulatory controls. Active travel strategies encompassed infrastructure expansion types (e.g. strategic cycleways, pedestrian networks), traffic reduction measures (e.g., Low Traffic Neighbourhoods, 20mph zones), and behaviour-change programmes.

Greenspace intervention types prioritised tree planting, habitat restoration and biodiversity net gain measures, alongside hazard control functions (ecosystem services) such as cooling, flood storage, and air quality buffering. Heat risk management and responses were comparatively narrower consisting of urban cooling measures, retrofit approaches to reduce overheating, passive design measures in new builds, and heatwave preparedness mechanisms (e.g., cool spaces, early warning systems). Food strategies focused primarily on food waste reduction, community food growing initiatives and food insecurity responses, with fewer explicit adaptation oriented actions. Overall, infrastructure and regulatory interventions were most developed in housing, flooding, air quality and transport sectors, while heat and food systems focused more on green infrastructure, resilience and behavioural interventions.

### Cross-sectoral strategy alignment and policy integration

#### Strong alignment across policy documents

**Fig. 4:**
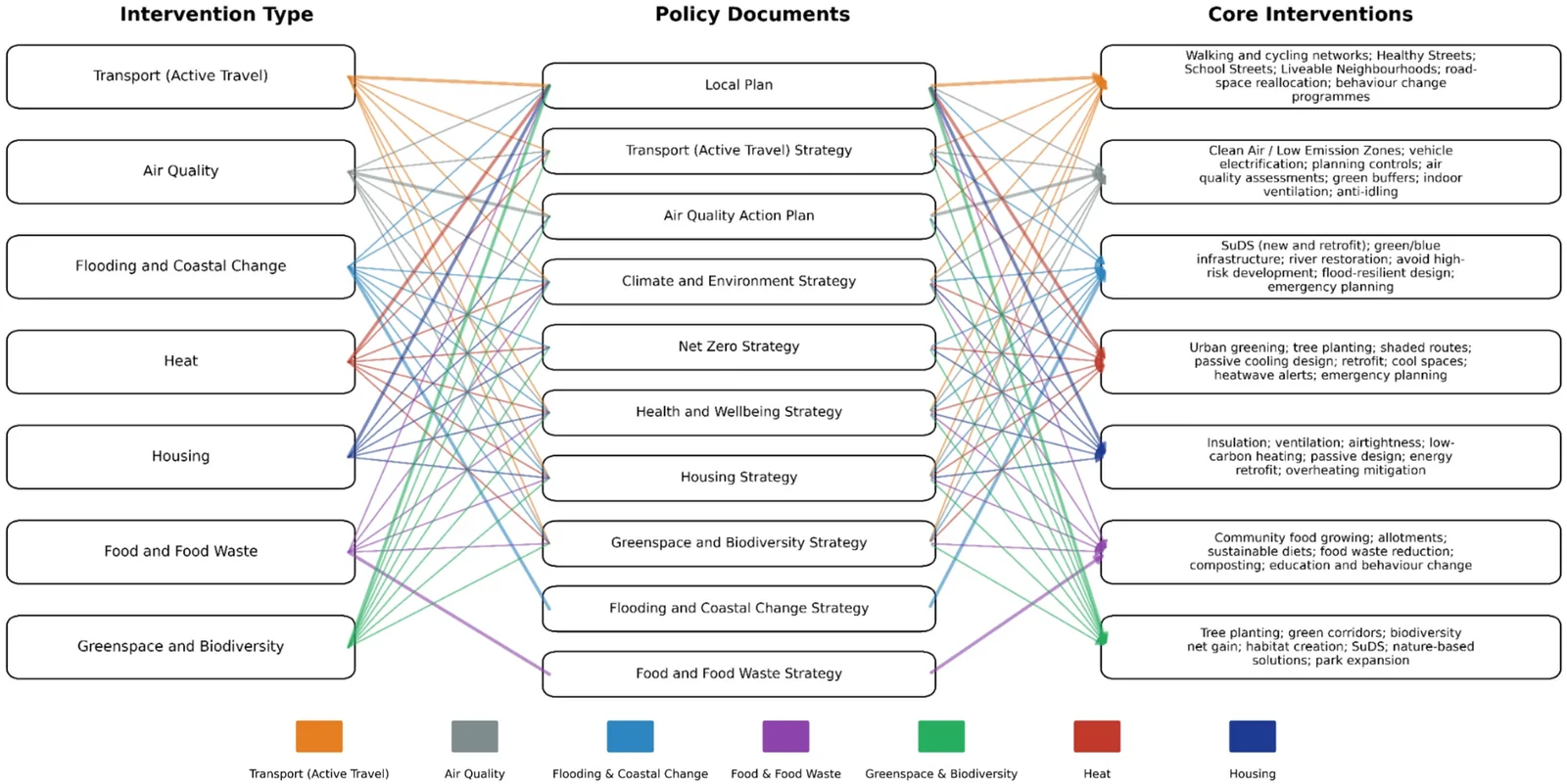
Alignment of climate–health interventions across policy documents. The diagram shows how intervention types (left) are connected to local authority policy documents (centre) and associated intervention areas (right). Coloured connectors indicate alignment, illustrating the integrated and multi-sector nature of local climate and health action. Bold lines are the lead contributor in each pathway

Several intervention areas demonstrated relatively strong cross-sectoral alignment across local authority policy documents (Fig. 4). Active travel, housing retrofit, flood resilience and greenspace interventions were consistently referenced across multiple policy domains and were commonly framed as contributing to both climate and public health objectives. These interventions frequently appeared within climate action plans, local and corporate plans, housing strategies, transport strategies, air quality action plans and health and wellbeing strategies, although the rationale and framing varied between sectors.

Active travel was one of the most consistently referenced interventions. Walking, wheeling and cycling infrastructure, healthy streets approaches, school streets and traffic reduction measures were widely promoted as mechanisms for reducing transport emissions, improving air quality and increasing physical activity. However, while transport, climate and air quality strategies commonly identified these co-benefits, consideration of climate resilience measures such as shaded routes, flood-resilient infrastructure and sustainable drainage integrated into streetscapes was less consistently incorporated across health and wellbeing strategies.

Housing interventions also demonstrated substantial alignment across policy documents. Energy efficiency improvements, low-carbon heating systems, improved ventilation and climate-resilient building design were frequently framed as contributing to emissions reduction while also addressing fuel poverty, respiratory health, damp and mould, excess winter mortality and thermal comfort. Local Plans typically provided the regulatory framework for implementation, while climate and net zero strategies reinforced technical standards and housing and health strategies more explicitly identified vulnerable populations likely to benefit from interventions.

Flood resilience was similarly characterised by strong integration across policy domains. Avoidance of development in high-risk flood areas, implementation of Sustainable Drainage Systems (SuDS), natural flood management approaches and nature-based solutions appeared across planning, housing, climate, transport and flood risk management documents. These interventions were often framed as providing multiple co-benefits, including reduced flood risk, improved environmental quality, increased access to greenspace and enhanced community resilience.

Greenspace and biodiversity interventions also demonstrated substantial alignment. Tree planting, habitat restoration, biodiversity net gain, green roofs and wider green infrastructure initiatives were embedded across planning, climate, housing, transport and air quality strategies. These interventions were associated with multiple climate-health objectives, including urban cooling, air quality improvement, flood mitigation, physical activity promotion and mental wellbeing.

No local authority included in this review had a standalone heat strategy or comprehensive heat-health action plan. However, heat-related interventions were identified across multiple policy documents, including climate, planning, housing, transport, biodiversity and public health strategies. Common areas of alignment included urban greening, tree planting, green corridors, passive building design, housing retrofit and measures to reduce indoor overheating. Climate, public health and net zero strategies were more likely to identify vulnerable populations and propose targeted actions such as cool spaces, heatwave alerts and adaptations within care settings, whereas Local Plans and infrastructure strategies typically addressed heat indirectly through development standards and urban design requirements.

**Fig. 5:**
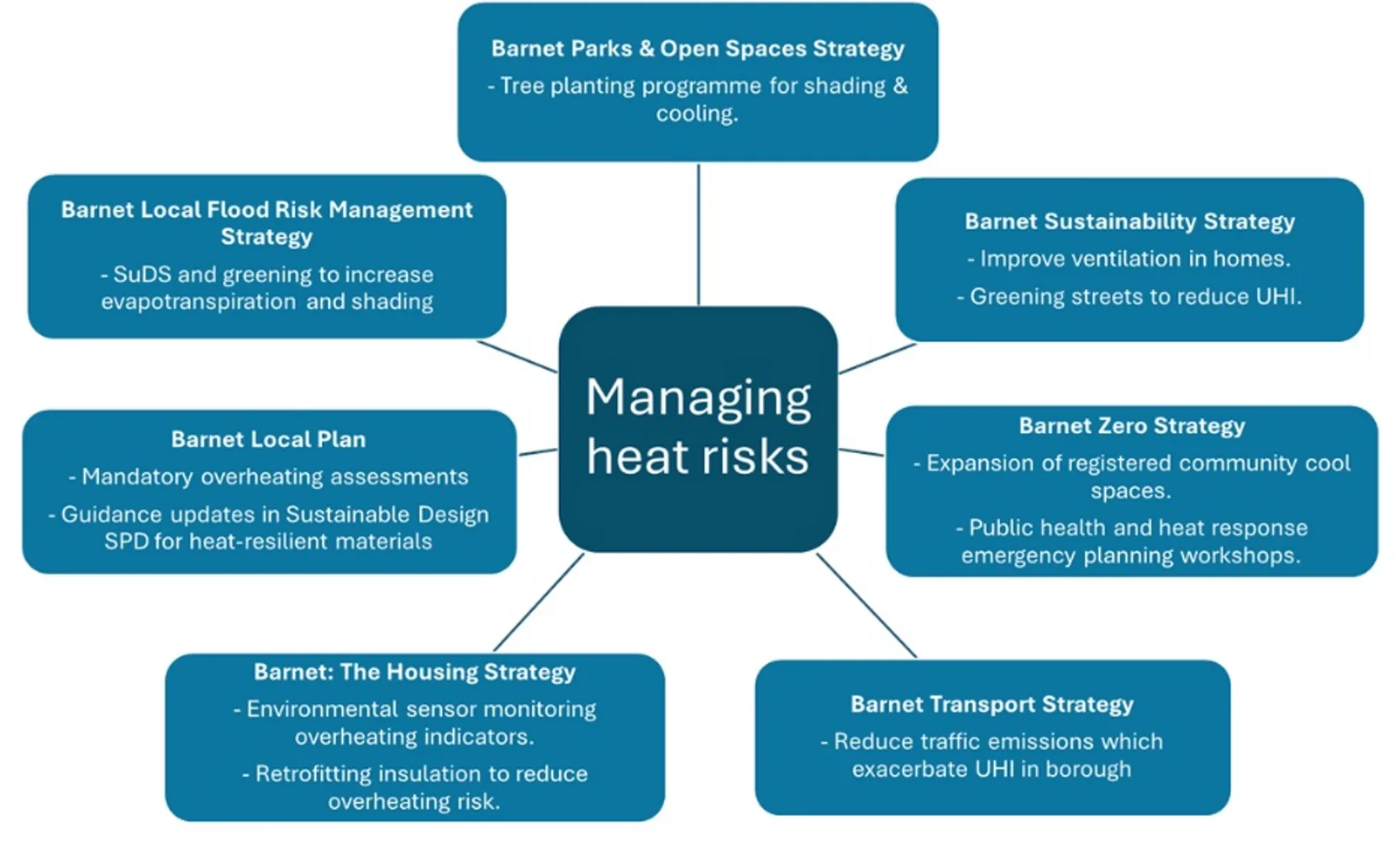
Illustrative example of local authority (Barnet) policy documents contributing to the management of heat risks, showing how policies across planning, housing, transport, sustainability, flood risk, and open spaces interact to address overheating and urban heat

Figure 5 illustrates how multiple policy documents within the London Borough of Barnet contribute to the management of heat-related health risks. Immediate response measures include cool spaces and emergency planning; medium-term adaptation actions include housing retrofit and shading interventions; and longer-term prevention approaches focus on sustainable development, urban greening and climate-resilient design. This example demonstrates how heat risk management is distributed across multiple sectors and policy documents and the lack of a dedicated heat action plan does not necessarily imply lack of implementation of heat risk management measures.

#### Inconsistencies and gaps in alignment

Food systems demonstrated little integration across policy documents. Although food-related interventions were recognised as relevant to both health and climate objectives, they were unevenly distributed across strategies and often lacked explicit cross-referencing between policy areas. Community food growing, food insecurity responses, sustainable diets and food waste reduction were most commonly identified within food strategies, with more limited integration into wider climate, planning and public health documents. This pattern may partly reflect the functional responsibilities of different policy domains, as food interventions are not equally relevant across all sectors.

#### Implementation mechanisms

The extent to which policy documents described implementation mechanisms varied considerably across sectors and document types. Operational strategies, including Air Quality Action Plans, Local Flood Risk Management Strategies and transport plans, were more likely to include delivery plans, implementation timelines, partnership arrangements and monitoring frameworks. In contrast, climate action plans and broader corporate strategies typically established strategic priorities and objectives while relying on sector-specific policies for implementation.

Partnership working was a common implementation mechanism across policy domains. Many strategies identified collaboration between local authority departments, Integrated Care Systems, transport authorities, housing providers, environmental organisations and community groups as important for delivering climate and health objectives. Cross-referencing between policy documents was also frequently used to demonstrate alignment with wider local priorities and statutory responsibilities.

Monitoring and evaluation arrangements were less consistently developed. While many strategies included indicators relating to emissions reduction, transport use, housing performance or environmental quality, relatively few incorporated health-specific outcome measures. Explicit monitoring of health inequalities, vulnerability reduction or climate-related health outcomes was uncommon. As a result, although many policy documents identified interventions with potential health benefits, mechanisms for assessing their health impacts were often less clearly articulated.

#### Synergies and tensions

Many interventions were framed as delivering synergies between climate mitigation, adaptation and public health objectives. Active travel interventions were frequently associated with emissions reduction, improved air quality and increased physical activity, while greenspace interventions were linked to urban cooling, biodiversity enhancement, flood mitigation and mental wellbeing. Housing retrofit programmes similarly combined carbon reduction objectives with improvements in thermal comfort and respiratory health.

Potential tensions and trade-offs were discussed less frequently. Where identified, these included balancing transport network changes with accessibility and road safety considerations, managing potential overheating risks associated with highly energy-efficient buildings, and balancing competing land-use priorities associated with housing development, flood management and biodiversity objectives. Overall, policy documents more commonly emphasised co-benefits than potential conflicts.

### Vulnerability and inequalities

Recognition of climate change impacts on health inequality was widespread across policy documents, particularly in relation to deprivation, fuel poverty, and older populations, yet few strategies translate these issues into clearly targeted interventions. Heat vulnerability is less prioritised in comparison to winter fuel poverty which is more often treated as an immediate risk. Across strategies, the most consistently identified vulnerable groups are older people, children, low-income households, residents of poor quality housing, individuals with chronic illness, and socially marginalised communities. Air quality and transport policies demonstrate the most systematic integration of health inequalities, explicitly linking deprivation, ethnicity, roadside exposure, and pre-existing respiratory or cardiovascular disease. Housing interventions similarly embed strong equity framing, particularly around fuel poverty, damp, mould, ventilation, and poor thermal conditions, while heat adaptation strategies recognise unequal vulnerability but reference ethnicity less consistently.

In contrast, greenspace, flooding, and food system interventions tend to address inequalities more indirectly. Overall, children, older adults, low-income communities, and residents of dense roadside neighbourhoods are the most consistently prioritised groups, while ethnic minority and intersectional vulnerabilities receive strongest attention in air quality and transport domains. Inequality framing appears most explicit where environmental exposures are direct and measurable, and more implicit where benefits are broader across populations.

## Discussion

### Summary of key findings.

This study provides descriptive evidence on how local authorities in England frame public health priorities climate actions across policy documents and how interventions are integrated across sectors relevant to population health. While the review identifies areas of stronger and weaker policy integration, it does not assess implementation effectiveness, resource allocation or the extent to which interventions improve health outcomes (or reduce carbon emissions) in practice. An important finding was the degree of alignment across objectives and policy documents around several intervention types; particularly active travel, housing retrofit, greenspace access and quality and flood risk management. However, while many interventions have clear potential to improve population health, public health is mentioned in half of the policy documents reviewed but not commonly positioned as a primary driver of local climate action. Instead, public health outcomes are typically framed as a secondary benefit of environmental or climate interventions, with responsibilities dispersed across multiple policy domains and limited mechanisms for coordination or evaluation. Existing literature examining local climate action plans have similarly found that health considerations are inconsistently integrated within climate strategies and often appear as ancillary benefits rather than explicit policy objectives [5, 9, 25]. This study demonstrates how interventions that shape major determinants of health are already distributed across sector-specific policies but lack explicit public health framing, coordination, and evaluation. The results therefore suggest that advancing climate and public health integration may depend less on creating new strategies and more on strengthening climate and health objectives, embedding public health expertise, aligning interventions across existing policy domains and development of integrated evaluation plans to ensure outcomes are measured.

### Public health integration within local climate governance.

The limited prominence of public health within local climate action and sectoral strategies may partly reflect the governance context within which climate policy is developed in local government. Studies have found that while many local authorities recognise the health implications of climate change, health considerations are often unevenly integrated into climate strategies and adaptation planning [5, 9]. Framing climate action more explicitly through public health outcomes may therefore strengthen policy prioritisation and communication, while helping to articulate the broader social benefits of climate action. Global analysis of city mitigation actions further demonstrates that although urban climate interventions can deliver substantial health co-benefits, awareness and reporting of these benefits varies widely across sectors [26]. While over three-quarters of UK local authorities have declared climate emergencies and committed to ambitious climate targets, this review indicates that implementation frameworks, delivery models and funding arrangements vary considerably across authorities dependent on the hazard or sector [10], therefore translating these commitments into coordinated policy action remains challenging [27]. Very few policy documents outlined specific monitoring mechanisms for intervention implementation and implementation commitments are predominantly relied on to be delivered through sector-specific strategies. Through consideration or prioritisation of public health outcomes in responding to local climate risks, integrating economic return on investment analysis may strengthen an economic argument for investment in climate mitigation or adaptation [28].

The institutional position of public health within local government may further contribute to these dynamics. Following the transfer of public health responsibilities from the National Health Service to local authorities in 2013, public health teams were placed within a governance environment intended to support action on the wider determinants of health. However, integration across policy domains has been uneven across councils [29]. Qualitative research with public health professionals working in English local authorities suggests that many recognise climate change as a major public health issue but report uncertainty about their role, limited capacity and insufficient institutional support for climate-related work [30]. While the COVID-19 pandemic increased the visibility of public health expertise within local government, this review found variation in the extent to which public health objectives were explicitly incorporated across local climate-related policy documents.

The challenges identified in this review are not unique to England. Studies from other UK nations and OECD countries have similarly reported difficulties integrating climate adaptation, mitigation and public health objectives across local government structures^(31)^. Although governance arrangements and statutory responsibilities differ internationally, fragmentation between sectors, limited institutional capacity and variable incorporation of health considerations within climate planning appear to be common challenges. These findings therefore contribute to a wider international discussion regarding multisectoral climate-health policy integration.

### Behavioural approaches and structural policy pathways

The analysis also highlights an important distinction between behavioural and structural approaches within local strategies. Many interventions identified in this study, particularly within active travel and food policy, focus on encouraging behavioural change among individuals, for example through promoting walking and cycling or supporting community food initiatives. While such initiatives can generate important local benefits, evidence from climate governance and public health research suggests that behaviour-focused interventions are most effective when supported by structural changes that shape the environments in which behaviours occur [31, 32]; and while behaviour change interventions can have impacts at the individual level they rarely have significant outcomes at the population level [33]. Structural policy mechanisms including planning policies that prioritise active travel infrastructure, energy efficient housing standards, and urban design interventions that increase access to greenspace; offer greater potential for sustained improvements in both climate outcomes and population health. This distinction is particularly relevant as research on healthy urban environments has emphasised the importance of planning systems in shaping exposure to environmental risks and supporting healthier living conditions [24, 34]. The findings of this study suggest that planning and housing policies may represent particularly important pathways for delivering long-term climate and public health co-benefits.

### Addressing health inequalities 

Although vulnerable populations were frequently identified across policy documents, particularly older adults, children, low-income households and people with chronic health conditions, these references were not consistently translated into targeted interventions or measurable equity objectives. In many cases, inequalities were acknowledged as an important consideration but were not systematically incorporated into implementation, monitoring or evaluation frameworks. This finding is important given evidence that climate-related health risks are distributed unequally and may exacerbate existing social and environmental inequalities.

A further implication of the findings concerns the relationship between climate policy and health inequalities. While some strategies reference vulnerable populations, relatively few systematically assess the distributional impacts of climate interventions or evaluate how policies might reduce or exacerbate health inequalities. This is significant given the strong links between climate risks and the social determinants of health such as housing quality, income, and neighbourhood environments. Recent feasibility research demonstrates that decision support tools, such as vulnerability mapping that integrates hazard exposure with social and health vulnerability indices, can enhance local adaptation planning [35, 36]. Local authority capacity may also shape the scale and effectiveness of local climate action as differences in UK climate governance highlights substantial variation in institutional resources and capabilities between authorities [17]. These differences may shape both the scale, effectiveness and distribution of benefits from local interventions across communities. Since 2010, austerity-driven reductions in central government funding have significantly reshaped the financial capacity of local authorities, contributing to increasing geographical inequalities in local government resources and service provision [37]. Such structural constraints may influence the extent to which local authorities can develop and implement ambitious climate and public health interventions.

### Cross-sectoral alignment and implementation mechanisms

The findings suggest that challenges relating to policy coherence extend beyond individual sectors. While active travel, housing retrofit and greenspace interventions were frequently integrated across multiple policy documents, other areas such as food systems and heat adaptation demonstrated more fragmented approaches. These differences partly reflect the varying statutory responsibilities and functional boundaries of policy sectors, but also highlight the ongoing challenge of embedding climate-health objectives consistently across local authority planning processes. The likelihood of more compound climate events in the future may lead to cascading impacts on critical services including health care delivery [38]. Adaptation to extreme heat illustrates this dynamic particularly clearly. Although none of the local authorities reviewed had a dedicated heat strategy, a range of relevant interventions, including urban greening, passive cooling design, housing retrofit and the provision of cool spaces, were embedded across multiple strategies. This suggests that heat risk management is often addressed indirectly through sectoral policies rather than through a single dedicated strategy. Importantly, this does not necessarily indicate a lack of action. Instead, effective management of heat risks may depend more on the alignment of sectoral interventions and the integration of public health considerations within existing policies. Evaluation methods of heat plans in Arizona highlighted a lack of alignment and cross-referencing across strategy types, for example a disconnect between hazard mitigation plans and other land use or climate plans in heat exposure and vulnerability [39]. Given that heat exposure varies geographically across England, strengthening cross-sector coordination may provide a more flexible approach to managing climate-related health risks than developing additional hazard-specific strategies.

In practice, many of the interventions identified are also shaped by national policy targets and sectoral regulatory frameworks, such as housing standards, planning guidance and air quality requirements, which influence local authority action even when policies are not explicitly framed around climate or health objectives. The role of national mandated requirements to ensure local authorities protect individuals from the impacts of extreme weather and climate change is repeatedly recognised as a powerful and necessary policy lever [8, 40]. This reflects a broader challenge of policy integration within urban climate governance, where mitigation, adaptation and public health objectives are frequently pursued through sector specific policy instruments rather than through fully integrated strategies [41]. Strengthening coordination across these policy domains therefore represents an important opportunity to maximise the health benefits of climate action while capitalising on existing policy levers within local governance systems.

A notable finding was the variation in implementation detail across policy documents. Operational plans, particularly flood risk management, transport and air quality strategies, were more likely to include delivery mechanisms, partnership arrangements and monitoring frameworks than broader climate strategies. However, explicit monitoring of climate-related health outcomes was uncommon. This may limit opportunities to evaluate whether interventions achieve intended health benefits and reduce climate-related vulnerabilities over time.

### Strengths and limitations

This study provides a systematic analysis of local authority policy documents across multiple sectors, offering insight into how climate and public health objectives are represented within local governance frameworks. By examining strategies spanning housing, transport, greenspace, food systems and environmental policy, the analysis captures the cross-sectoral nature of climate interventions and identifies areas of both alignment and fragmentation across policy domains. This study has several limitations. First, the review included 12 local authorities from four regions of England and was designed to capture variation in geography, governance structures and socio-demographic characteristics rather than provide a nationally representative sample. Consequently, findings may not reflect the full diversity of climate-health policy approaches across all English local authorities. Second, the analysis focused on policy documents and therefore reflects how climate-health priorities are articulated within formal policy frameworks rather than how they are implemented in practice. The study was unable to assess organisational culture, political leadership, resource allocation, partnership working or intervention effectiveness, all of which are likely to influence climate-health integration and outcomes. Future research could build on these findings through comparative case studies, interviews with policy actors and implementation-focused evaluations to explore how stronger climate-health integration is achieved in practice, alignment between local climate risks and intervention prioritisation and which organisational factors support successful cross-sector collaboration.

## Conclusions

Local authorities across England are implementing a wide range of interventions with potential climate and public health benefits. However, the extent to which climate-health objectives are explicitly integrated across policy documents varies between sectors, with stronger alignment observed in areas such as active travel, housing retrofit, greenspace and flood resilience than in food systems and heat adaptation. While vulnerable populations are frequently recognised, equity considerations are not always translated into targeted action or monitoring frameworks. Strengthening cross-sector coordination, implementation mechanisms and evaluation of health outcomes may help local authorities maximise climate-health co-benefits and reduce climate-related health inequalities.

These findings suggest that the key challenge for local climate and health policy coherence is not the absence of relevant interventions but the need to better integrate public health within their design, coordination and monitoring. Strengthening cross-sector collaboration and embedding public health within climate decision-making may help local governments maximise health benefits while addressing place-based environmental health risks and inequalities.

## Supplementary Information

Supplementary Material 1.

## Data Availability

The datasets used and/or analysed during the current study are available from the corresponding author on reasonable request.
