# Supplementary material for: How are local authorities responding to climate change and health impacts? a policy document analysis of four England regions
Source: BMC Public Health. 2026 Jul 31;26:3087. doi: 10.1186/s12889-026-28752-9 (PMC13711811; doi:10.1186/s12889-026-28752-9)
Supplement: Supplementary file 1 [file 12889_2026_28752_MOESM1_ESM.docx]

**Supplementary material: Local Authority climate and health interventions**

# **S1: Interventions by sector**

## **S1.1. Transport (active travel) interventions**

| **Strategy** | **Interventions** |
| --- | --- |
| Local plan | - Promotion and expansion of walking, wheeling and cycling, including Healthy Route Networks and citywide active travel strategies. - Expansion and enhancement of walking and cycling networks (e.g. Barnet Loop, Bee Network, Leaway, Greenway, Cycle Superhighways, Great North Cycleway). - Healthy Streets–led design, including quieter, less polluted routes, improved public realm, and 20 mph zones. - Reduction of car dominance and dependency through modal shift, particularly in town centres and neighbourhoods. - Measures to reduce short car journeys and encourage active and sustainable travel choices. - Safe routes to school and strong School Travel Plans. - Active travel routes integrated into new developments and high-accessibility development locations to reduce car use. - Improved public transport accessibility and connectivity, including enhanced orbital and radial links. - Investment in public transport, including integrated networks, Mass Rapid Transit safeguarding, and lobbying for network extensions (e.g. Metrolink). - Use of cleaner and more sustainable transport technologies, including cleaner engines and reduced emissions from commercial and public sector vehicles. - Infrastructure for electric vehicles, including EV charging points and updated cycle parking standards. - Reduced transport emissions and air quality improvements linked to transport design and logistics management. - Consideration of COVID-19-related opportunities to accelerate active travel uptake. - Improved pedestrian and cycling connectivity, including enhanced east–west links, crossings, and access to green spaces. - Use of waterways (e.g. River Thames) for freight and passenger transport to reduce road traffic and emissions. |
| Climate or Environment Strategy | - Reduce private car use and congestion through modal shift, particularly for short journeys. - Develop 15-minute neighbourhoods to reduce car dependency and support local access. - Expand and invest in walking, wheeling and cycling infrastructure and networks. - Implement low-traffic and car-lite streets, including Low Traffic Neighbourhoods and School Streets. - Apply Healthy / Streets-for-All approaches to reallocate road space for people, greening, SuDS and safer streets. - Improve public transport accessibility, reliability and connectivity, including bus, rapid transit and rail investment. - Support integrated transport systems prioritising walking, cycling and public transport. - Promote active and sustainable travel behaviour change, including bike hire, e-bikes, training and cycle-to-work schemes. - Enable transition to low- and zero-emission vehicles, including EV charging, hydrogen buses and fleet electrification. - Promote sustainable freight and reduce emissions from commercial and public-sector vehicles. - Improve air quality through reduced transport emissions, supported by monitoring and Clean Air Plan delivery. - Integrate active travel and transport hierarchy principles into planning policy and new development. - Enhance access to green and open spaces via active travel networks to support healthier lifestyles. - Design climate resilience and adaptation into transport infrastructure (heat, flooding, storms) |
| Net Zero or low carbon strategy | - Deliver new and expanded cycling and walking routes, including strategic networks and local connections - Improve accessibility and inclusivity of public transport, including step-free access at stations. - Achieve modal shift to walking, cycling and public transport to reduce congestion, noise and air pollution. - Reduce dependence on private cars through demand management and road-space reallocation to active and low-carbon modes. - Support 15-minute neighbourhoods by locating services, jobs and facilities close to homes and accessible by sustainable transport. - Expand low- and zero-emission transport infrastructure, including EV charging (on-street and off-street), hydrogen transport and ULEV buses. - Implement Healthy Streets / Healthy Pupil and public-realm improvements to support safer, more attractive walking and cycling environments. - Enhance town centre and local centre public spaces to prioritise pedestrians, cyclists and public transport. - Integrate transport and land-use planning through Local Transport Plans and Cycling & Walking Infrastructure Plans. |
| Housing | - Invest in cycling infrastructure and support measures, including secure cycle storage and cycle training, with targeted support for lower-income residents. - Integrate estates and neighbourhoods into low-traffic environments, Healthy Streets, and safe, connected walking and cycling routes. - Require new development to follow Healthy Streets principles, low-traffic design, and air-quality-positive layouts. - Improve public realm and green infrastructure to encourage walking and cycling and reduce exposure to traffic pollution. - Prioritise development in locations with high accessibility by public transport, walking and cycling networks. - Deliver sustainable, connected neighbourhoods integrating walkability, public transport and low-carbon mobility. - Apply planning policies that require safe, continuous pedestrian and cycle connections within and beyond developments. - Support public transport provision through development planning. - Reduce car dependency to support modal shift, climate mitigation and improved air quality. |
| Food & food waste | n/a |
| Flooding & coastal change | - Protect critical transport infrastructure (roads, bridges, rail and tram networks) from flood risk. - Improve highway drainage to reduce surface-water flooding, including gully maintenance and upgrades in high-risk areas. - Integrate SuDS into highways and street design to manage runoff and reduce flood risk (e.g. rain gardens, verge SuDS, highway runoff schemes). - Combine flood-resilient street design with traffic calming and low-traffic neighbourhood schemes. - Use multifunctional spaces (e.g. car parks, public realm) to store shallow floodwater safely during extreme rainfall. - Work in partnership with infrastructure providers (e.g. National Highways) to explore natural flood management and improve network resilience. - Maintain and operate transport and drainage assets to keep roads and walking/cycling routes passable during flood events. - Identify transport vulnerabilities and rapid-response catchments, ensuring safe and accessible emergency routes. - Apply lessons from past storm events and Transport Resilience Reviews to strengthen transport system resilience. - Use smart sensors, asset mapping and community-led programmes (e.g. Operation Watershed) to monitor and manage drainage systems. |
| Transport (active travel) | - Expand borough- and region-wide walking, wheeling and cycling networks, including segregated cycleways and strategic routes. - Deliver Healthy Streets / Liveable Neighbourhoods, including low-traffic neighbourhoods, road-space reallocation and rat-run removal. - Improve road safety through Vision Zero / Safe Systems approaches, including 20 mph zones, safer junctions, crossings and casualty-reduction schemes. - Roll out School Streets and child-friendly street measures to reduce traffic and improve air quality around schools. - Enhance public realm and accessibility, including dropped kerbs, tactile paving, lighting, wayfinding and secure cycle parking. - Improve integration between active travel and public transport, including step-free access, safe routes to stations, mobility hubs and real-time information. - Promote modal shift away from private cars to active and public transport to reduce emissions, improve air quality and address the climate emergency. - Support behaviour-change programmes and smarter travel choices, including training, education, marketing and employer-based initiatives. - Introduce and support micromobility (e-bikes, scooters, water taxis) to improve access and reduce emissions. - Design active travel infrastructure to be climate-ready, providing shaded, flood-resilient, safe and comfortable routes. |
| Greenspace or biodiversity strategy | - Expand walking and cycling networks through green corridors, greenways and linear open spaces - Improve connectivity between parks, neighbourhoods, workplaces and town centres via active travel routes. - Deliver strategic recreational and commuter routes (e.g. Barnet Loop and other greenway links). - Enhance Public Rights of Way to support safe, accessible non-car travel. - Integrate walking and cycling routes with green infrastructure and greenspace policies. - Create tree-lined, shaded and pleasant routes to support cooling, air quality and climate adaptation. - Reduce exposure to traffic pollution by routing active travel through green, low-traffic environments. - Embed active travel and green connectivity requirements within planning policy to support modal shift, reduced car use and improved health. |
| Air quality action plan | - Expand walking, wheeling and cycling networks, including segregated cycleways, strategic routes, local improvements, and School Streets. - Develop cycle hire schemes, micromobility (e-bikes, scooters), and cargo bike/logistics hubs to support short trips and last-mile deliveries. - Provide secure cycle parking, wayfinding, lighting, and safe, accessible connections to public transport. - Integrate active travel into planning, commissioning, and behaviour-change programmes (school/employee initiatives, “smarter travel” campaigns). - Improve bus and rail accessibility, frequency, reliability, and emissions standards (e.g., BRT, Park & Ride, Northumberland Line, Superroutes). - Introduce priority lanes, traffic signal coordination, high-occupancy vehicle lanes, and integrated travel apps to support modal shift. - Support multi-modal connections linking active travel with public transport hubs. - Expand EV charging infrastructure, on-street and off-street, including rapid chargers. - Transition council, bus, taxi, and freight fleets to low- or zero-emission vehicles (ULEV, hydrogen, Euro VI standards). - Support car clubs, car sharing, and taxi licensing policies to promote low-emission travel. - Implement road-space reallocation, congestion management, parking reforms (CPZs), and Low Emission Zones / Ultra-Low Emission Zones - Encourage mode shift from private cars through behavioural programmes, travel planning, and reduced school-run traffic initiatives. - Traffic calming, speed reductions, and street redesigns to enhance safety and reduce car dominance. - Anti-idling campaigns around schools, stations, and hubs to reduce local pollution. - Route active travel through green corridors and low-pollution areas where possible. - Promote healthier, low-emission streets through shading, greening, and public realm improvements. - Integrate active travel and low-carbon transport strategies to support climate mitigation and resilience. |
| Health & wellbeing strategy | - Expand walking, cycling, and wheeling infrastructure, including segregated routes, Healthy Routes, Low Traffic Neighbourhoods, and School Streets. - Rebalance kerbside and public space use through the Kerbside Strategy and traffic-calming interventions. - Promote physical activity and sustainable travel through programmes like Active Lambeth Strategy, GM Moving, and Local Delivery Pilots. - Reduce transport emissions, improve air quality, and support low-emission transport options, including EV charging and low-carbon vehicles. - Ensure active travel and sports/leisure infrastructure is safe, accessible, inclusive, and designed with sustainability and climate resilience in mind. - Increase connectivity to services, amenities, and public transport while providing affordable and accessible transport options, including pilots for vulnerable groups. - Promote sustainable travel behaviours through communications, travel planning, education, and policy support to strengthen funding bids and uptake. |

## **S1.2. Air quality interventions**

| **Strategy** | **Interventions** |
| --- | --- |
| Local plan | - Reduce traffic-related emissions through active travel, sustainable transport policies, and promotion of walking, cycling, public transport, and cleaner freight strategies. - Promote public and low-carbon transport, including cleaner buses and low-emission vehicles. - Reduce emissions from council operations, including electrification of fleets, reduced travel, and building decarbonisation. - Encourage retrofitting of commercial and residential premises to lower emissions. - Implement policies and regulations to control industrial, development-related, odour, and other pollutant emissions. - Plant trees and enhance green spaces, including green high streets, to absorb particulate and gaseous pollution. - Require mandatory Air Quality Assessments for major developments, and sometimes minor developments. - Ensure new developments are Air Quality Neutral and comply with air quality planning policies (e.g., PH1, QOP2, EN15). - Restrict or mitigate uses that worsen pollution in Air Quality Management Areas (AQMAs) and implement measures to protect sensitive uses. - Promote compliance with Air Quality Action Plans, strategic frameworks, and relevant environmental strategies (e.g., London Environment Strategy). - Integrate air quality considerations into planning and development, including mitigation of exposure and monitoring of indoor and residential amenity - Implement district heating networks and clean energy solutions that protect air quality. - Monitor air quality through indicators and frameworks, triggering remedial action if air quality declines. - Produce stronger air quality action plans where needed and incorporate air quality improvement into climate and environmental priorities. - Work to ensure everyone can live in a healthy environment with clean air. |
| Climate or Environment Strategy | - Reduce emissions from transport, buildings, domestic burning, waste processes, and industrial sources. - Promote active travel and modal shift from private cars to walking, cycling, and public transport. - Support EV transition, fleet electrification, and expansion of EV charging infrastructure. - Implement cleaner heating technologies, heat decarbonisation, and low-emission fuels. - Plant trees, green screens, and other green infrastructure to capture pollutants and improve street-level air quality. - Monitor air quality with hyperlocal sensors (e.g., near hospitals, nurseries) and publish daily or annual data. - Implement Clean Air Plans, including ULEZ-aligned measures, targeting NO₂ and PM reductions. - Reduce petrol and diesel dependency and encourage uptake of low-emission technologies. - Apply planning policies to ensure new developments do not worsen air quality, mitigate exposure for sensitive uses (residential, schools, hospitals), and use planning obligations to fund improvements. - Integrate air quality into public sector commissioning, social value frameworks, and strategic planning. - Educate the public through programmes such as Air Quality Health Champions and Clean Air Day. - Monitor environmental health impacts linked to air pollution and remedial action where needed. - Apply legislative tools such as the Clean Air Act and Environmental Permitting to regulate emissions. - Promote nature-based solutions, including trees, hedgerows, and greening streets, as part of pollution mitigation. - Engage businesses and communities in low-emission initiatives and air quality improvement schemes. |
| Net Zero or low carbon strategy | - Deploy EV charging infrastructure and support uptake of ultra-low-emission vehicles (ULEVs). - Develop active travel infrastructure to encourage walking, cycling, and modal shift from private cars. - Reduce transport emissions through decarbonisation of vehicles and implementation of Clean Air Zones or related interventions. - Expand renewable energy generation to reduce reliance on polluting energy sources. - Reduce machinery-related emissions through measures such as reduced mowing. - Implement business sustainability audits, targeting improvements in refrigeration, cooking, and ventilation to reduce emissions. - Strategically expand and manage green spaces to capture pollutants and improve air quality. - Apply planning policies (e.g., EN15) to ensure developments do not worsen air quality, mitigate exposure for sensitive uses (residential, schools, hospitals), and use planning obligations to fund air quality measures. - Support citizens in self-managing air quality risks through real-time air quality data, awareness of lower-pollution travel routes, and targeted interventions in areas of high pollution or deprivation. |
| Housing | - Plant trees and develop green infrastructure to reduce local air pollution and capture particulates. - Promote walking, cycling, and sustainable transport to reduce vehicle emissions and traffic-related pollution. - Improve housing and neighbourhood design through Healthy Streets, Air Quality Positive design, and sustainable urban planning to reduce transport- and development-related pollution - Retrofit homes and buildings to improve energy efficiency, insulation, and thermal performance, reducing reliance on gas and solid fuels and lowering indoor/outdoor emissions. - Decarbonise heating through low-carbon or renewable systems to reduce NOx and other combustion-related pollutants. - Enhance ventilation systems and enforce high-quality ventilation standards (e.g., Future Homes Standard, Building Regulations) to improve indoor air quality. - Address damp and mould in homes as a respiratory health risk. - Use Sustainable Drainage Systems (SuDS), planting, and green infrastructure to reduce particulate pollution. - Conduct air quality assessments for new developments that may worsen emissions. - Integrate transport planning to minimise traffic generation and associated air pollution. - Apply water and energy efficiency retrofits to reduce emissions from homes. - Enforce housing standards to mitigate indoor pollutants and maintain healthy indoor environments. |
| Food & food waste | n/a |
| Flooding & coastal change | - Increase vegetation and tree cover to capture particulate pollution and improve local air quality. - Use Sustainable Drainage Systems (SuDS) to filter particulates and reduce pollution from surface runoff. - Reduce runoff and combined sewer overflow events through water-quality and drainage measures, delivering indirect air-quality benefits. - Deliver naturalisation and green infrastructure projects that reduce dust, limit degradation of built infrastructure, and support air-quality co-benefits. - Align green and water-management interventions with wider air-quality objectives, including Just Transition and climate-health goals. |
| Transport (active travel) | - Expand, enforce, and integrate Ultra Low Emission Zones (ULEZ), Clean Air Zones (CAZs), and Low Emission Zones to phase out the most polluting vehicles. - Reduce vehicle emissions through modal shift from private cars to walking, cycling, public transport, and rail. - Electrify buses, taxis, council fleets, and other public-sector vehicles, including zero-emission bus deployment in partnership with transport authorities. - Support uptake of ultra-low-emission vehicles through expanded EV charging infrastructure, including regionwide charging, EV-only lanes, and green taxi initiatives. - Implement cleaner freight, logistics, and construction standards, including freight consolidation, routing reforms, and NRMM emissions controls. - Enforce anti-idling measures, particularly outside schools and at congestion hotspots. - Reduce congestion and emissions through demand management, improved traffic flow, junction redesign, bus priority, and rail capacity improvements. - Redesign kerbside space and streets to prioritise low-emission mobility and reduce exposure to roadside pollution. - Target pollution hotspots and strategic roads through focused interventions, including collaboration with highways authorities. - Reduce traffic and exposure near schools and other sensitive locations. - Implement emissions-based parking, charging zones, and other measures to accelerate the phase-out of polluting vehicles. - Deliver behaviour-change and smart travel programmes to reduce single-occupancy car use, particularly in Air Quality Management Areas (AQMAs). - Align transport interventions with Air Quality Action Plans, Clean Air Plans, and AQMA management measures. - Use air quality monitoring, real-world emissions testing, and system upgrades to inform and evaluate transport and clean air interventions. |
| Greenspace or biodiversity strategy | - Expand tree planting and vegetation cover to filter airborne pollutants and improve local air quality. - Target tree planting, green infrastructure, and buffers along major roads and traffic corridors to reduce NO₂ and particulate exposure. - Develop and manage green corridors, meadows, wetlands, and parks to absorb pollutants and support respiratory health. - Use Sustainable Drainage Systems (SuDS), wetlands, and natural flood management to intercept pollutants and reduce polluted runoff. - Integrate air-quality benefits into development design through species selection, street-level planting, and urban greening standards. - Design greenspaces to act as buffers against air and noise pollution and to improve overall environmental quality. - Promote active travel through green travel corridors and well-connected parks, reducing reliance on car travel and traffic emissions. - Manage habitats and green infrastructure to deliver wider environmental co-benefits, including reductions in noise and light pollution. |
| Air quality action plan | - Expand and maintain a comprehensive air quality monitoring network, including PM₂.₅/PM₁₀ sensors, diffusion tubes, mobile and IoT monitoring, and school-based monitors. - Provide advanced air quality information services, including real-time public alerts (SMS/email), construction emissions alerts, and targeted warnings for vulnerable groups. - Use monitoring data to inform Air Quality Action Plans, AQMA management, public health needs assessments, and evaluation of interventions. - Enforce Smoke Control Areas and solid fuel regulations to reduce particulate pollution. - Regulate construction emissions through NRMM enforcement, dust monitoring, low-emission construction equipment, logistics controls, and application of IAQM dust guidance via planning conditions. - Require Air Quality Assessments and Air Quality Neutral/Positive standards in planning applications, including management of cumulative development impacts. - Develop and apply planning policy to reduce emissions during and after construction, including delivery emissions and operational impacts. - Consider and appraise Clean Air Zones and Low Emission Strategies, including health and economic impact assessments. - Enforce anti-idling legislation and promote compliance in high-exposure locations. - Encourage uptake of low- and zero-emission vehicles, including council fleets, taxis, and private hire vehicles, supported by EV charging infrastructure. - Apply tighter emission and age standards through taxi and private hire licensing - Regulate industrial emissions through Environmental Permitting and liaison with the Environment Agency. - Work with freight operators through Freight Quality Partnerships, route management, and consolidation centres to reduce emissions. - Promote travel planning, demand management, and parking policy changes to support modal shift and EV uptake - Integrate green infrastructure into air quality and climate adaptation strategies to support pollutant reduction. - Deliver public engagement, education, and behaviour-change campaigns (e.g. anti-idling, CleanBurn, school programmes, indoor air quality awareness) - Support indoor air quality improvement through campaigns addressing damp, mould, and wood burning. - Collaborate with schools, health services (GPs, hospitals), businesses, developers, BIDs, transport authorities, and neighbouring areas to deliver coordinated action. - Develop Local Area Energy Planning to phase out fossil fuel heating and reduce combustion-related emissions. |
| Health & wellbeing strategy | - Implement and regularly update Air Quality Action Plans to reduce emissions and population exposure. - Strengthen smoke control and tobacco control measures to reduce indoor and outdoor air pollution. - Align Local Plans, Transport Strategies, and climate programmes to support sustainable travel and low-emission vehicles. - Advocate for stronger national and regional air quality standards and regulatory frameworks. - Reduce traffic exposure for vulnerable communities by managing traffic, reducing the need to travel, and prioritising low-emission transport. - Apply spatial planning to locate schools, care homes, play areas, and other sensitive uses away from traffic pollution hotspots. - Use vegetation planting, green infrastructure, and natural buffers to reduce exposure to air pollution. - Engage large businesses and institutions through climate or clean-air agreements to reduce emissions (e.g. Climate Contracts, anchor-institution approaches) - Support health-system actions that reduce air pollution and improve respiratory outcomes, including decarbonisation of clinical practices (e.g. inhaler switching). - Train health professionals to identify, prevent, and support air-pollution-related health needs - Establish and support cross-authority air quality partnerships to coordinate action, share data, and deliver joint programmes. - Deliver public information and awareness campaigns on air quality and health, targeted at communities, professionals, and decision-makers. - Integrate air quality into planning processes, including provision of EV charging, differential parking charges, and development-led mitigation. - Use air quality needs assessments and monitoring data to inform local strategies, priorities, and evaluation of interventions. - Encourage sustainable practices across public-sector and NHS organisations, including low-emission travel, energy and resource efficiency, and waste reduction. - Undertake research to assess the public-health impacts of Air Quality Management Areas and guide targeted action. |

## **S1.3. Flooding & coastal change interventions**

| **Strategy** | **Interventions** |
| --- | --- |
| Local plan | - Application of Sequential and Exception Tests to steer development away from high flood risk areas, including avoidance of Flood Zone 3b except for water-compatible or essential infrastructure. - Mandatory site-specific Flood Risk Assessments for development in flood risk zones. - Restrictions on inappropriate development in the highest-risk locations unless regeneration need clearly outweighs flood risk. - Monitoring and refusal of developments that conflict with Environment Agency advice. - Requirement for safe access, egress, and emergency evacuation routes during flood events. - Limitations or prohibitions on residential basements in higher flood risk zones. - Raised floor levels above climate-change–adjusted flood levels. - Flood-resilient construction, including locating vulnerable uses above ground floor level. - Design-led flood mitigation integrated into site layout and public realm. - Future-proofing development to account for increased frequency and severity of extreme storm events. - Universal requirement for Sustainable Drainage Systems (SuDS), prioritising above-ground and nature-based solutions. - Use of greenfield runoff rates, brownfield runoff reductions, and climate change allowances in drainage design. - On-site surface water storage designed for extreme rainfall events and system exceedance. - Drainage Impact Assessments for major developments. - Integration of permeable surfaces, infiltration systems, and discharge hierarchies to manage runoff and avoid downstream flood risk. - Long-term maintenance arrangements for SuDS and drainage infrastructure. - Protection of groundwater and Source Protection Zones - Investment in green and blue infrastructure to reduce runoff, manage heat, and improve water quality. - River and watercourse restoration, including de-culverting where feasible. - Set-backs and easements from flood defences and watercourses to allow maintenance and future upgrades. - Strategic surface water storage facilities and flood mitigation infrastructure. - Enhancement of waterways, drainage networks, and natural flood management features. - Protection, maintenance, and improvement of flood defences, ensuring development does not compromise their effectiveness. - Integration of upgraded flood defences into high-quality public realm where possible. - Requirement for developer contributions to flood defence improvements where necessary. - Protection and enhancement of coastlines to maintain safety and resilience to climate change. - Coastal mitigation measures to protect designated sites affected by climate and recreational pressures. - Strategic flood mitigation in major growth and regeneration areas. - Place-based flood resilience projects, including innovation schemes, sponge parks, river corridor enhancements, and tree planting. - Integrated water management across developments and neighbourhoods. - Partnership working with water companies, the Environment Agency, and other risk management authorities. - Strengthened contingency and emergency planning for flooding, storms, and water stress. - Community engagement in flood risk management and resilience building. |
| Climate or Environment Strategy | - Expansion and implementation of Sustainable Drainage Systems (SuDS), including rain gardens, permeable pavements, green streets, green roofs, and water retention features. - Integration of SuDS into all major developments, with encouragement for smaller schemes, in line with national technical standards. - Use of greenfield or reduced runoff rates, infiltration, and surface water attenuation to manage rainfall without increasing flood risk elsewhere. - Enhancement and management of parks, green spaces, and public realm to absorb rainfall, store surface water, and reduce flood peaks. - Natural flood management and nature-based solutions, including wetland restoration, woodland creation, tree planting, peatland and upland restoration. - Investment in natural capital to improve water retention, manage runoff, and reduce surface water and fluvial flooding. - River catchment and catchment-wide water management approaches to address flooding from all sources. - Protection and restoration of wetlands, woodlands, nature reserves, and other habitats that contribute to flood mitigation. - Integration of biodiversity enhancement and biodiversity net gain with flood risk reduction measures. - Use of green and blue infrastructure strategies to manage urban hydrology and improve resilience to extreme rainfall. - Climate vulnerability mapping, flood risk mapping, and use of risk databases and typologies to identify hotspots and inform planning and infrastructure decisions. - Strategic flood risk assessment frameworks to guide development and flood mitigation at local and regional scales. - Flood-resilient and adaptive design principles embedded in planning policy, accounting for long-term flood and coastal change. - Avoidance of development in high flood risk areas where possible, applying Sequential and Exception Tests in line with national policy - Management of surface water, ordinary watercourses, and drainage systems under Lead Local Flood Authority responsibilities. - Improvement of flood resilience infrastructure alongside natural and nature-based measures. - Community flood resilience initiatives, including flood preparedness, awareness campaigns, resilience hubs, and support for property flood resilience and insurance uptake. - Integration of flood risk management with emergency planning and multi-agency response arrangements. - Water efficiency, rainwater harvesting, and greywater reuse to reduce pressure on drainage systems. - Protection of communities, businesses, and critical services from flooding through coordinated flood risk management strategies. - Coastal and shoreline management to address erosion and flood risk linked to climate change. - Alignment of flood risk management actions with Local Flood Risk Management Strategies, climate adaptation plans, and Local Nature Recovery Strategies. |
| Net Zero or low carbon strategy | - Deliver major Sustainable Drainage Systems (SuDS), including wetlands, rain gardens, swales, tree pits, green roofs, and green walls, to manage surface water and reduce flood risk. - Require all major developments to incorporate SuDS in line with national technical standards, with smaller developments encouraged to do so where feasible. - Design SuDS to achieve greenfield runoff rates wherever possible, avoid increased contamination risk, and maximise ecological, landscape, and tree-planting benefits. - Integrate natural flood management measures into new and existing developments to slow runoff, reduce peak flows, and enhance biodiversity and carbon sequestration. - Retrofit green infrastructure into existing urban areas, including the removal of impermeable surfaces in gardens, driveways, and public spaces. - Enhance and replace temporary flood defences with permanent, climate-resilient infrastructure in vulnerable locations, including city centres and waterfronts. - Deliver targeted flood-risk reduction schemes in high-risk locations, prioritising protection for vulnerable households and deprived communities. - Invest in drainage infrastructure to reduce surface water flooding and improve system capacity under future climate scenarios. - Use Strategic Flood Risk Assessments and Local Flood Risk Management Plans to guide land use planning, investment decisions, and mitigation measures. - Direct development to areas at lowest flood risk through the Sequential and Exception Tests, taking account of climate change and all sources of flooding. - Ensure development in areas of flood risk does not increase flood risk elsewhere or reduce floodplain storage capacity. - Incorporate embedded carbon assessment into the design and delivery of flood defence and drainage infrastructure. - Improve water quality through pollution-reducing planting and nature-based solutions integrated with flood management schemes. - Develop long-term flood adaptation plans informed by the latest UK climate projections, covering fluvial, surface water, groundwater, and coastal risks. - Raise awareness of flood risk through updated flood mapping, public engagement, and education programmes. - Increase uptake of flood warning and alert services for homes and businesses in areas at risk. - Support household and business preparedness through guidance on property-level resilience and flood response actions. - Maintain and regularly test multi-agency emergency response arrangements, including Major Incident Plans for flood events. - Strengthen collaboration with Local Resilience Forums and partner organisations to improve emergency preparedness and community resilience. - Ensure health and social care facilities have appropriate flood risk plans, emergency capacity, and arrangements to minimise health impacts during flood events. - Deliver flood risk management through partnership working, securing external funding and aligning interventions with wider climate adaptation and regeneration objectives. |
| Housing | - Embed flood resilience and water management as a core consideration in spatial planning, housing delivery, and regeneration. - Direct new development to locations at lowest flood risk through plan-led site selection and risk-based decision-making. - Integrate sustainable drainage and green infrastructure as standard practice across new development and regeneration. - Reduce surface water flooding through catchment-based approaches, including natural flood management and upstream interventions. - Protect, restore, and naturalise watercourses, floodplains, and river corridors to enhance flood storage, biodiversity, and water quality. - Improve the resilience of existing homes and communities through property-level flood adaptation and retrofit programmes. - Ensure all new council-led housing is designed to be flood resilient and climate-ready. - Address flood risk to vulnerable people, communities, and land uses through targeted adaptation and, where necessary, relocation. - Improve surface and groundwater quality by reducing diffuse pollution from development, highways, and urban areas. - Align flood risk management with biodiversity net gain, nutrient neutrality, and wider nature recovery objectives. - Strengthen long-term resilience through climate-informed planning, using UK Climate Risk Assessment evidence and future climate projections. - Support regeneration in coastal and flood-prone areas in ways that reduce deprivation while increasing climate resilience. - Maintain effective emergency preparedness and recovery arrangements to reduce the impacts of flood events. - Deliver flood risk management through partnership working across planning, housing, infrastructure, and environmental bodies. |
| Food & food waste | - Promote community planting that helps biodiversity and flood alleviation. - Food system resilience planning for supply disruptions. - Use of green infrastructure to increase water absorption and reduce runoff. |
| Flooding & coastal change | - Identify, map, and prioritise flood risk areas using up-to-date modelling, historic evidence, and risk assessments, including Surface Water Management Plans, Strategic Flood Risk Assessments, and Preliminary Flood Risk Assessments. - Apply catchment-based flood risk management, integrating surface water, fluvial, and coastal processes through coordinated planning and delivery. - Deliver Sustainable Drainage Systems (SuDS) across new development and through retrofit programmes, including green roofs, detention basins, swales, ponds, and rain gardens. - Retrofit SuDS and blue–green infrastructure into highways, public spaces, parks, and estates to manage surface water at source. - Implement Natural Flood Management measures, including woodland planting, wetland creation, river re-meandering, upstream storage, and land management changes to slow flows and reduce flood peaks. - Restore, naturalise, and maintain rivers, ordinary watercourses, and culverts to improve conveyance, storage, ecological condition, and resilience. - Manage overland flow routes and exceedance pathways through land use planning, green infrastructure, and surface water attenuation features. - Improve drainage and sewerage capacity through targeted investment, including separation of surface water from foul systems and collaboration with water companies. - Maintain, upgrade, and enhance flood defences, including tidal barriers, pumping stations, storage areas, and coastal protection assets, using a risk-based and climate-resilient approach. - Implement Shoreline Management Plan and Catchment Flood Management Plan policies, including hold-the-line, managed realignment, or no active intervention where appropriate. - Deliver targeted flood alleviation schemes in priority risk areas, informed by wet-spot analysis and local flood investigations - Develop, maintain, and regularly update asset registers identifying structures and systems that affect flood risk, including ownership and maintenance responsibilities. - Improve inspection, maintenance, and performance of drainage assets, including highway gullies, trash screens, ditches, and culverts. - Use real-time monitoring, sensors, and forecasting to improve understanding of system performance and flood risk. - Incorporate climate change allowances and cumulative development impacts into flood modelling, planning decisions, and infrastructure design. - Ensure planning and development management controls prevent increased flood risk and safeguard overland flow routes, flood storage, and watercourse corridors. - Strengthen flood warning services, public communication, and community engagement to improve preparedness and response. - Support and coordinate multi-agency emergency planning, flood response, and recovery arrangements. - Protect and improve the resilience of critical infrastructure, including power, transport, water, and telecommunications assets. - Collaborate with the Environment Agency, water companies, coastal authorities, and neighbouring councils on joint delivery of fluvial, surface water, and coastal schemes. - Promote nature-based and blue–green infrastructure solutions that deliver flood risk reduction alongside biodiversity, water quality, and amenity benefits. |
| Transport (active travel) | - Embed flood resilience and climate adaptation into the planning, design, and operation of transport networks. - Reduce flood risk and surface water impacts on streets and transport corridors through sustainable drainage and blue–green infrastructure. - Design streets and neighbourhoods to manage water naturally and minimise disruption to movement and access during heavy rainfall. - Improve the resilience of road, rail, and bridge assets to flooding and extreme weather. - Maintain and adapt highways and transport infrastructure using risk-based, climate-informed approaches. - Protect access to critical services, rural communities, coastal areas, and international gateways during flood events. - Incorporate nature-based and non-hard engineering solutions into transport schemes wherever feasible. - Use innovation, monitoring, and data to improve understanding of flood risk and asset performance across transport networks. - Future-proof transport infrastructure by accounting for climate change impacts in investment and design decisions. - Deliver flood-resilient transport through coordinated planning, maintenance, and partnership working. |
| Greenspace or biodiversity strategy | - Integrate Sustainable Drainage Systems (SuDS) into urban spaces, streets, gardens, parks, cycleways, and development sites to manage surface water and reduce flood risk. - Retrofit SuDS and blue–green infrastructure into existing public and amenity spaces, including parks, wetlands, and open spaces - Restore, naturalise, and de-culvert rivers and watercourses to improve conveyance, flood storage, and ecological function. - Enhance river corridors, floodplains, and waterways to absorb rainfall, slow flows, reduce downstream flooding, and support biodiversity. - Create and maintain wetlands, flood storage areas, and aquatic green spaces as hydrological assets that provide adaptation, cooling, and wildlife benefits. - Apply natural flood management techniques, including tree and vegetation planting, riverbank reinforcement, upstream storage, and flow attenuation measures. - Use soft landscaping, depaving, and other permeable surfaces to reduce surface runoff in residential, commercial, and public spaces. - Ensure protection and no net loss of functional water-mitigating land uses, such as parks, wetlands, and flood storage areas, in planning and capital programmes. - Design greenspaces and open areas to mitigate climate change impacts while supporting flood risk management, carbon storage, and ecosystem services. - Integrate water-sensitive urban design and flood-alleviation principles into all relevant capital, regeneration, and development programmes. |
| Air quality action plan | - n/a |
| Health & wellbeing strategy | - Avoid development in areas at high flood risk and manage new development to minimise exposure. - Respond proactively to sea level rise, increased rainfall, and surface water flooding through planning and infrastructure measures. - Integrate Sustainable Drainage Systems (SuDS) into development and urban spaces, including water harvesting, permeable surfaces, and wastewater recycling. - Incorporate green and blue infrastructure to manage stormwater, reduce runoff, and enhance ecological and community benefits - Ensure adaptation and flood risk considerations are embedded across planning, housing, and environmental programmes. - Promote safe and resilient built environments through cross-sector partnership working and strategic adaptation planning. |

## **S1.4 Food & food waste interventions**

| **Strategy** | **Interventions** |
| --- | --- |
| Local plan | - Promote reuse, reduce, repair, and recycling across all sectors. - Encourage waste minimisation through design in developments, including space for composting and recycling. - Facilitate reuse and recycling of construction materials. - Support community reuse initiatives (e.g., community skips schemes) to reduce fly-tipping. - Align waste management targets with local and regional waste policies. - Support sustainable waste disposal choices for households and businesses. - Invest in sustainable waste management infrastructure to reduce emissions from transport and processing. - Ensure on-site waste facilities in developments to improve hygiene and environmental standards. - Develop local waste reprocessing and energy production facilities to support a closed-loop economy. - Strengthen waste and recycling services to improve environmental quality. - Support community food growing, allotments, gardens, and orchards for food security, health, and wellbeing. - Encourage temporary or underutilised land (e.g., vacant plots, green roofs, open spaces) for local food growing. - Incorporate productive plants and trees in landscaping schemes to enhance local food production. - Protect local shops and markets to ensure access to healthy, locally produced food, reducing travel emissions. - Support sustainable land-based food production sectors, including agri-tech and water management. - Partner with food redistribution groups (e.g., FareShare) to reduce food waste and improve access to healthy food. - Strengthen community responses to food insecurity, particularly in vulnerable populations. - Restrict hot food takeaways near schools and other areas linked to poor dietary health. - Incorporate health impact assessments (HIA) for planning policies affecting food access and dietary health. - Provide education programs for businesses and individuals on waste and sustainable food practices. - Strategies should aim to reduce emissions and environmental impact while supporting local resilience and wellbeing. |
| Climate or Environment Strategy | - Promote local food growing for resilience, community cohesion, physical activity, and mental health. - Support community gardens, allotments, and local food production to increase food security and access. - Expand food-waste collection, composting, and waste-reduction programmes for households and businesses. - Reduce food waste through redistribution to those in need and behaviour-change campaigns (e.g., Love Food, Hate Waste). - Implement circular economy approaches: reuse, repair, recycling, and reduction of bulky and single-use waste. - Upgrade communal and kerbside waste facilities, including glass recycling, to improve coverage in dense or deprived areas. - Reduce environmental impacts of waste: plastic pollution, vermin, contamination, and methane emissions from landfill. - Promote sustainable diets consistent with healthy and climate-friendly guidelines (e.g., EAT-Lancet) to improve nutrition and reduce emissions. - Strengthen local food supply chains and community resilience to reduce vulnerability to global shocks. - Support education and engagement: schools, VCSEs, and local residents on food security, sustainability, and waste reduction. - Enhance natural resource protection by reducing consumption, reusing organic residues, and returning nutrients to soil. - Encourage sustainable agricultural and land management practices to improve local food system resilience and reduce carbon. |
| Net Zero or low carbon strategy | - Support community food growing, gardens, and local food sites to increase food security, resilience, and educational opportunities. - Provide training and educational programmes on food growing, sustainable diets, and basic cookery skills. - Promote sustainable food initiatives through local partnerships to encourage climate-friendly diets and lower-carbon food sourcing. - Reduce food waste at household and community levels through separation, composting, redistribution, and behaviour-change programmes. - Support repair cafés and community reuse schemes to minimise waste and promote a circular economy. - Encourage local food sourcing to reduce supply-chain emissions and improve local food security. - Embed circular economy and waste-reduction targets into local policies and infrastructure projects. - Provide guidance for caterers and individuals on sustainable food choices, child nutrition, and low-carbon diets. - Incorporate climate-friendly considerations into building and flood defence designs that affect food systems. |
| Housing | - Support food-growing spaces and community gardens on housing estates and within neighbourhoods. - Integrate community food growing into green infrastructure, contributing to climate resilience and food security. - Create and manage biodiverse spaces that improve soil health, ecosystem services, and local food resilience. - Require adequate refuse storage, recycling, and waste management provision in new developments to support sustainable waste practices. |
| Food & food waste | - Promote sustainable, healthy, and climate-friendly diets, including plant-rich options, across communities, schools, early years, and public-sector catering. - Strengthen local and sustainable food procurement, including shorter supply chains, food hubs, dynamic procurement, and increased sourcing from local producers. - Support local food production, including urban and community growing, protection of high-quality agricultural land, repurposing underused spaces, and support for small-scale commercial farms. - Expand community food growing, gardens, and allotments, including training for residents and professionals to build skills and resilience. - Reduce food insecurity by tackling underlying causes, supporting sustainable food clubs and social supermarkets, and improving uptake of welfare schemes (e.g. Healthy Start). - Strengthen the resilience and sustainability of third-sector food aid providers, shifting from emergency aid toward long-term food resilience. - Reduce food waste across households, retail, hospitality, and public sectors through behaviour-change campaigns, business standards, and education. - Expand food-waste collection services, surplus food redistribution, and use of digital platforms (e.g. Olio, Too Good To Go). - Promote Love Food Hate Waste–style campaigns, practical cooking initiatives (e.g. one-pot and slow-cooker projects), and business engagement on waste reduction - Support circular economy approaches to food and waste, including composting, redistribution, and returning organic material to soils. - Improve waste and recycling systems linked to food production and consumption, reducing landfill, emissions, and environmental impacts. - Encourage agroecological, regenerative, and nature-positive farming practices, including soil health, woodland, peat, and nature recovery. - Support local food businesses and entrepreneurs through training, business support, partnerships, and promotion of sustainable food standards and awards. - Promote fair, ethical, and sustainable food policies, including Fairtrade, sustainable fish sourcing, and improved public-sector food standards. - Increase access to healthy, affordable food by reducing food deserts, supporting local retailers and markets, and promoting local shopping. - Build a more resilient food system that reduces reliance on long, vulnerable supply chains and strengthens local capacity to withstand climate and economic shocks. |
| Flooding & coastal change | - Waste management improvements via trash-screen upgrades to prevent blockages - Removal of concrete and naturalisation of watercourses to improve water quality (important for food safety where allotments are near watercourses) - Protection of agricultural land through catchment-based flood management. - Manage sewer flooding risk to reduce contamination. - Natural flood management (NFM) improves soil, water retention and reduces runoff. |
| Transport (active travel) | - Freight consolidation to reduce HGV trips. - Sustainable freight operations and construction vehicle standards. - Consolidated waste collection to reduce traffic, emissions and air pollution - Encourage co-ordinated waste and recycling collection in new developments - Micro-distribution for more local deliveries, enabling low-emission modes |
| Greenspace or biodiversity strategy | - Expand community food-growing sites, including allotments, orchards, and edible landscapes, to support food security, biodiversity, and climate resilience. - Protect allotments and community gardens as key green infrastructure assets supporting local food production and resilience - Integrate food growing into parks and green spaces, recognising social, health, and wellbeing benefits. - Improve management, security, and quality of allotment sites, including increased self-management and effective waiting-list management. - Create new allotments and growing spaces where demand is high to improve access to local food growing. - Promote biodiverse gardens and green spaces, supporting pollinators, soil health, and ecosystem services. - Use green waste and meadow/grassland management to support circular use of organic material and enhance soil quality. - Recognise allotment provision as part of local recreation standards, embedding food growing within place-based planning. - Support waste recycling and organic material reuse linked to green infrastructure and food-growing spaces. |
| Air quality action plan | - Regulate emissions from commercial cooking, including controls on solid-fuel and charcoal cooking, to reduce PM₂.₅ and protect air quality - Enforce smoke control and statutory nuisance powers to address emissions, odour, and combustion from commercial premises - Promote garden waste collection services to reduce bonfire burning and associated air pollution. - Plan and implement transition of waste and council vehicle fleets to lower-emission alternatives, reducing fuel use and transport emissions. - Reduce emissions from refuse collection, service vehicles, and council fleets through cleaner vehicles and operational efficiency improvements. |
| Health & wellbeing strategy | - Deliver Food Poverty and Insecurity Action Plans to reduce health inequalities and strengthen food system resilience. - Increase uptake of Healthy Start vouchers, free school meals, and holiday food programmes, particularly in communities facing food insecurity. - Target inequalities in access to good food, with a focus on Black, Asian and minority ethnic households and other disproportionately affected groups. - Promote sustainable, low-carbon and healthy diets through place-based dietary frameworks and public-sector leadership. - Support food businesses to reduce food waste and packaging, increase local procurement, and expand plant-based options. - Expand community food-growing spaces, including grey-to-green interventions, to support climate-resilient diets and wellbeing. - Use planning and licensing powers to shape healthier food environments, including reducing the density of unhealthy food outlets. - Implement food security and poverty-reduction measures to protect health during climate-related food price volatility and shocks. - Support sustainable waste management, including space for recycling and composting in developments. - Safeguard land for waste management facilities and reduce the environmental impacts of waste. - Monitor foodbank usage and drivers of food insecurity to inform targeted, preventative interventions. |

## **S1.5. Greenspace & biodiversity interventions**

| **Strategy** | **Interventions** |
| --- | --- |
| Local plan | - Embed green infrastructure and greenspace at the heart of the physical environment through planning and development - Develop and implement Parks & Open Spaces Strategies and Local Nature Recovery Strategies. - Protect existing open spaces through a no net loss principle, alongside quality and accessibility improvements. - Require biodiversity net gain (minimum 10%) for developments, proportionate to scale. - Integrate green infrastructure into new developments, including green roofs and walls, street trees and sustainable drainage. - Make significant and sustained investment in parks and open spaces, including long-term maintenance. - Expand, improve and invest in large networks of parks and open spaces. - Improve the quality, safety, inclusivity and accessibility of green spaces. - Address Areas of Park Deficiency to ensure equitable access across neighbourhoods. - Support high-quality play parks, outdoor sport and leisure facilities, and informal recreation - Maintain recognised quality standards for parks and green spaces (e.g. Green Flag). - Deliver ambitious, long-term tree-planting programmes across streets, parks and new woodlands - Protect existing trees, with clear replacement and woodland management plans. - Increase urban tree canopy cover to support cooling, air quality improvement and biodiversity. - Conserve, restore and enhance priority habitats and species, including designated and locally important sites - Enhance biodiversity across parks, woodlands, playing fields and semi-natural spaces. - Create, strengthen and connect habitat networks to reduce fragmentation and increase resilience. - Implement long-term monitoring and management of newly created habitats. - Protect, expand and enhance strategic green infrastructure networks and green grids. - Improve connectivity through green corridors, waterways, river valleys and urban–rural links. - Improve access to rivers and waterways while safeguarding ecological integrity. - Use nature-based solutions to support flood risk management, sustainable drainage and climate resilience. - Reduce urban heat island effects through expanded greenspace and canopy cover. - Improve air quality through strategic planting and green infrastructure. - Explicitly recognise open spaces as climate adaptation and natural capital assets. - Ensure all neighbourhoods have access to high-quality green and natural spaces. - Improve access to parks, allotments and community gardens to support health and wellbeing. - Work with communities to co-design, deliver and steward local green projects. - Prioritise investment and access improvements in urban, deprived and high-need areas. |
| Climate or Environment Strategy | - Increase and diversify greenspaces, including parks, woodlands, meadows, nature reserves, green roofs, living walls, and blue infrastructure. - Deliver major tree-planting and canopy expansion programmes, including street trees, community tree initiatives, and large-scale woodland schemes. - Restore and create habitats such as wetlands, peatlands, grasslands, and wildlife areas to enhance biodiversity and ecosystem resilience. - Implement biodiversity net gain, habitat connectivity, and Local Nature Recovery Strategies to strengthen ecological networks. - Use nature-based solutions for climate adaptation, including flood management, heat mitigation, sustainable drainage, and air quality improvement. - Protect and enhance existing green networks, wildlife sites, and designated areas, ensuring development does not fragment habitats. - Improve access to greenspaces, parks, and natural areas to support mental and physical health, wellbeing, and community engagement. - Promote sustainable land management and ecological enhancements, including rewilding, pollinator corridors, and habitat-friendly landscaping. |
| Net Zero or low carbon strategy | - Implement large- and small-scale tree-planting programmes, including street trees, Tiny Forests, orchards, and community tree initiatives, to increase canopy cover, provide shading, and support air quality. - Expand and enhance greenspaces, including parks, meadows, wildlife areas, school grounds, river and estuary edges, and other natural corridors. - Restore and create habitats to improve biodiversity, ecosystem resilience, carbon storage, and climate adaptation. - Use nature-based solutions such as flood-absorbing landscapes, extended tree cover, sustainable planting, and wildlife-friendly landscaping. - Protect, consolidate, and extend existing green networks, Local Green Spaces, wildlife sites, and biodiversity priority areas, ensuring development does not fragment habitats and delivers net biodiversity gain. - Adjust land management practices to support carbon storage, biodiversity, and ecosystem services, including no-mow regimes, scrub regeneration, and conservation grasslands. - Promote community engagement, volunteering, and educational programmes to support nature recovery, climate resilience, and sustainable gardening practices. - Improve access to greenspaces and natural areas for health, wellbeing, and public use. |
| Housing | - Maintain and improve green and planted areas to high standards, including trees, gardens, and landscaped public realms. - Expand biodiversity on estates and neighbourhoods through tree planting, green roofs, climate-resilient habitats, and support for urban forest strategies. - Support community gardens, food-growing spaces, and opportunities for local engagement in greenspace management. - Integrate green infrastructure into new developments, neighbourhoods, and streetscapes to enhance multifunctionality, connectivity, and visual amenity. - Use green infrastructure and nature-based solutions to mitigate climate risks, including overheating, flood risk, and air pollution. - Protect and enhance existing green infrastructure, trees, and landscape features, ensuring developments do not fragment networks or harm ecological value. - Deliver biodiversity net gain through planning policies, ecosystem restoration, and collaboration with local nature partnerships. - Create attractive, connected, and walkable neighbourhoods with accessible green spaces, play areas, and improved public realm, promoting health, wellbeing, and social inclusion. |
| Food & food waste | - Expand and support community food-growing spaces, including allotments, gardens, orchards, and public land for growing. - Promote sustainable, regenerative, and agroecological practices in local food production to enhance biodiversity, soil health, and climate resilience. - Integrate food-growing opportunities into planning and housing policy to increase accessibility and community participation. - Encourage pollinator-friendly planting, seed-saving, and wildlife-friendly land management in community and agricultural spaces. - Build local resilience, skills, and environmental literacy through community gardening, food-growing projects, and stewardship programmes. - Map, monitor, and plan for community food-growing infrastructure, including the appointment of dedicated roles (e.g., Biodiversity Officer) and implementation of Biodiversity Net Gain plans. - Use green and community spaces for local food initiatives, events, and educational activities to strengthen sustainable local food systems and social engagement. |
| Flooding & coastal change | - Use parks, open spaces, and urban greenspaces for multi-functional purposes, including surface water storage, flood attenuation, carbon sequestration, and biodiversity enhancement. - Implement Sustainable Drainage Systems (SuDS), rain gardens, swales, wetlands, and other green/blue infrastructure to manage runoff, improve water quality, and support natural flood management. - Restore and naturalise rivers, riverbanks, and floodplains, including removal of hard channels, creation of wetlands, and re-establishing natural hydrological processes. - Integrate natural flood management with habitat creation, biodiversity net gain, and ecological connectivity, including wildlife corridors. - Protect designated and European protected nature conservation sites while implementing flood and water management measures. - Employ catchment-scale approaches, including tree planting, upstream storage, erosion control, and improved land management in agricultural and urban areas. - Align flood management and water-sensitive interventions with Local Nature Recovery Strategies, River Basin Management Plans, and broader biodiversity enhancement initiatives. - Engage communities in flood and habitat restoration projects to support stewardship, education, and co-benefits such as amenity and recreational use. |
| Transport (active travel) | - Implement street greening, pocket parks, green screens, and green roofs to enhance urban public realms, biodiversity, and climate resilience. - Deliver major tree-planting programmes, including large-scale initiatives like City of Trees, to improve air quality, reduce heat, manage flooding, and support wellbeing. - Integrate green and blue infrastructure into streets, public spaces, and transport corridors to support sustainable drainage, urban cooling, and habitat creation. - Improve walkability, active travel corridors, and access to green and historic spaces, linking mobility with health, climate adaptation, and environmental benefits. - Prioritise sustainable transport and reduce vehicle dominance to free space for greening and improve environmental quality. - Ensure infrastructure projects contribute to biodiversity net gain, habitat enhancement, and the protection of sensitive landscapes. - Incorporate nature-based solutions, wetlands, and other ecological features into urban infrastructure to maximize co-benefits for mental and physical health, flood management, and ecosystem resilience. |
| Greenspace or biodiversity strategy | - Enhance and expand parks, green spaces, local nature reserves, woodlands, and habitat corridors, including street trees and urban woodlands. - Strengthen ecological networks, wildlife corridors, and Local Wildlife Sites to improve biodiversity, connectivity, and climate resilience. - Restore, rewild, and naturalise habitats including meadows, wetlands, orchards, and pollinator areas, while managing invasive species and protecting ancient/priority woodlands. - Implement multifunctional greenspace designs to support climate adaptation (cooling, flood resilience, carbon storage), human health and wellbeing, amenity, and natural surveillance. - Increase tree canopy cover and plant new trees to reduce heat, improve air quality, provide shade, and support mental wellbeing. - Protect, maintain, and manage Sites of Special Scientific Interest, National Nature Reserves, Scheduled Monuments, and other designated ecological or heritage areas. - Apply nature-based solutions to improve pollination, water quality, air quality, and ecosystem services, including Sustainable Drainage Systems (SuDS). - Promote biodiversity net gain, natural capital accounting, and evidence-based planning to guide investment in under-served areas. - Improve accessibility to green and natural spaces for all residents, integrating inclusive play, youth engagement, community stewardship, and volunteering. - Support environmental education, citizen science, and public engagement programmes to foster sustainable behaviours, environmental literacy, and active participation in nature recovery. - Upgrade lower-quality parks and open spaces to safe, welcoming, and well-managed standards, including Green Flag recognition. |
| Air quality action plan | - Invest in and expand green infrastructure across urban areas, including parks, street trees, hedges, pocket parks, green walls, planters, and fresh-air squares. - Integrate green infrastructure into planning and development, ensuring connectivity across town centres, transport corridors, and neighbourhoods. - Use vegetation and green spaces to reduce pollution exposure, filter air pollutants (NO₂, PM, ozone), and improve local air quality. - Promote greening initiatives in Low Emission Neighbourhoods, schools, playgrounds, and public streets. - Support community engagement and stewardship through programmes like tree-planting partnerships, Playing Out traffic-free streets, and Tree Warden schemes. - Maintain and enhance existing green infrastructure to ensure long-term functionality, climate resilience, and public health benefits. - Align greening initiatives with wider policy frameworks, including climate action plans, environmental strategies, and Healthy Places design guidance. |
| Health & wellbeing strategy | - Increase tree planting and expand green infrastructure, including parks, parklets, and natural areas, to improve access, neighbourhood cooling, and flood resilience. - Enhance and create connected habitats, wildlife corridors, and biodiversity, ensuring net gain in all developments. - Improve access to parks, green spaces, and natural environments for all communities, promoting physical activity, wellbeing, and mental health resilience. - Integrate climate resilience and urban greening into planning, policy, and strategies, including grey-to-green initiatives and Just Transition actions. - Promote the health benefits of nature through green exercise, conservation activities, volunteering, and engagement with the historic environment. - Research, monitor, and communicate the impacts of nature-based interventions on health, wellbeing, and social care outcomes to guide future initiatives. - Protect and restore natural ecosystems while embedding multifunctional greenspaces that provide environmental, social, and climate co-benefits. |

## **S1.6. Heat interventions**

| **Strategy** | **Interventions** |
| --- | --- |
| Local plan | - Ensure new and existing homes meet high energy-efficiency and environmental standards. - Retrofit existing homes, including historic buildings, to improve insulation, heating, and thermal comfort. - Assess and mitigate overheating risks through orientation, shading, ventilation, dual-aspect layouts, thermal mass, and green/brown roofs. - Incorporate minimum internal space, accessible/adaptable layouts, and private/communal outdoor space to support health and wellbeing. - Locate developments away from flood- and pollution-prone areas and major roads. - Require flood-resilient construction, raised thresholds, safe access/egress, and sustainable drainage systems (SuDS). - Integrate green infrastructure, urban greening, tree planting, and parks to reduce heat island effects, improve air quality, and manage stormwater. - Promote low-carbon heat networks, sustainable materials, and net-zero/low-carbon construction. - Ensure large-scale housing and regeneration schemes integrate active travel routes, greenspace, and social infrastructure. - Improve private rented sector housing standards via licensing and enforcement. - Bring vacant properties back into use to reduce neighborhood decay and environmental hazards. - Address homelessness, overcrowding, and housing stability to reduce exposure to cold, heat, and poor-quality housing. - Require water efficiency, greywater/reuse systems, and climate-resilient building practices in new developments. - Ensure housing design is flexible for future adaptation to climate change and alternative uses. |
| Climate or Environment Strategy | - Expand urban tree canopy, shading, and green infrastructure to reduce heat exposure and mitigate urban heat islands. - Increase access to green and blue spaces (parks, water bodies) for cooling, recreation, and wellbeing. - Retrofit homes and public buildings to improve thermal comfort, insulation, ventilation, and reflective surfaces to reduce overheating. - Promote passive and climate-resilient design in new developments, including orientation, shading, natural ventilation, and green/blue roofs. - Implement permeable, shaded pavements and “cool corridors” to reduce local surface temperatures. - Use heatwave and overheating risk mapping, vulnerability assessments, and urban heat monitoring to inform planning and preparedness. - Develop and implement heatwave action plans, emergency response protocols, and public health awareness campaigns. - Integrate heat risk and adaptation into spatial planning, Local Plans, and climate adaptation strategies. - Target adaptation measures for vulnerable populations (e.g., older adults, pregnant women, schools, hospitals, care homes). - Encourage active travel and reduced vehicle use to lower heat-intensifying air pollution during extreme heat events. - Track and report heat-related risks and resilience measures through institutional monitoring and reporting frameworks. |
| Net Zero or low carbon strategy | - Expand access to publicly available Cool Spaces and air-conditioned areas for residents during heatwaves. - Integrate heat health alerts and the Heatwave Plan for England into local emergency planning and response. - Conduct heat resilience research with universities and partners to inform policy and practice. - Run workshops and training with Public Health, Emergency Planning, and health facilities on heat risk and preparedness. - Adapt hospitals, care homes, and other facilities to extreme heat (e.g., cool spaces, medicine storage, risk registers). - Increase tree canopy, green infrastructure, and natural shading to reduce urban heat islands and improve air quality. - Promote green walls and roofs on new and existing buildings, and shade for cycle and pedestrian routes. - Publicise heatwave resources, maps of Cool Spaces, and advice through libraries, media, and community networks. - Integrate nature-based solutions and man-made shading interventions to enhance cooling in public areas. - Prioritise heatwave and overheating considerations in upcoming Climate Adaptation Strategies. |
| Housing | - Incorporate shading solutions in homes and new developments, including trees, green roofs, green walls, canopies, curtains, blinds, and tinted/glass treatments. - Improve ventilation through natural (openable windows, passive design) and mechanical means (heat recovery, fans) to reduce indoor overheating. - Retrofit insulation, energy-efficient glazing, reflective materials, and building fabric upgrades to reduce heat gain and improve thermal comfort. - Promote low-energy, high-performance building standards (e.g., Passivhaus, Future Homes Standard) to minimise overheating in new builds. - Implement nature-based cooling and landscaping around homes and streets to mitigate urban heat island effects. - Monitor indoor temperatures and humidity with environmental sensors to identify overheating risks and inform interventions. - Address damp, mould, and condensation, which are linked to thermal extremes and poor indoor air quality. - Improve energy performance (EPCs) and energy management to stabilise indoor temperatures and enhance resilience for fuel-poor households. - Ensure accessible and adaptable housing designs consider heat vulnerability, particularly for older adults and other at-risk groups. - Require climate risk assessment in planning and development to identify and mitigate overheating risks. - Integrate microclimate mitigation, passive cooling, and shading policies into local planning and development standards. |
| Food & food waste | - Promote local resilient food production and regenerative agriculture to maintain productivity during heat extremes. - Use planting and urban greening to provide cooling benefits in cities. |
| Flooding & coastal change | - Implement tree-planting programs (e.g., 900/year) to provide shade and reduce urban heat island effects. - Expand green infrastructure, vegetation, and blue-green features to cool urban areas through shading and evapotranspiration. - Integrate Sustainable Drainage Systems (SuDS) to enhance shading, reduce surface heat, and support natural cooling. - Use Natural Flood Management (NFM) approaches and other greening strategies to increase overall green cover and improve thermal regulation. - Promote urban greening to reduce heat stored in hard surfaces and provide co-benefits for cooling, air quality, and wellbeing. |
| Transport (active travel) | - Increase shade and shelter along streets, including street trees, canopies, and other greening measures, to reduce urban heat exposure. - Plant and maintain street trees and urban greenery (1,000+ planned) to mitigate heat island effects and provide cooling. - Use resilient, climate-proof materials in streets and public infrastructure to withstand high temperatures. - Incorporate shade and cooling measures into walking and cycling routes, supporting active travel even during heat extremes. - Monitor surface and air temperatures with road sensors and digital tools to detect heat-related risks and inform early warning systems. - Climate-proof transport and electrification infrastructure to reduce heat-related pollution peaks and maintain network resilience during extreme heat. - Reduce traffic emissions and promote sustainable transport alternatives to minimise urban heat and exposure for pedestrians and cyclists. - Plan streets and infrastructure as part of Healthy Streets indicators to integrate shade, shelter, and climate adaptation into public spaces. |
| Greenspace or biodiversity strategy | - Increase tree canopy, woodland creation, and street/frontage planting to provide shade and reduce urban heat island effects. - Expand and enhance accessible parks, meadows, and semi-natural sites to serve as cooling refuges and support public health. - Develop and maintain green corridors to connect green spaces, ensuring cooling benefits reach dense urban neighbourhoods. - Incorporate blue-green infrastructure (wetlands, ponds, water features) to reduce local temperatures and provide shade. - Integrate green roofs, walls, and landscaping in buildings and developments to reduce heat gain and improve thermal comfort. - Ensure greenspace design and maintenance protects amenity and cooling benefits, including shaded seating and adapting to longer growing seasons. - Apply Urban Greening Factor or similar policies to require cooling features in new developments and promote nature-based urban heat mitigation. |
| Air quality action plan | - Retrofit buildings to improve energy efficiency and thermal comfort, reducing overheating and cold exposure. - Implement green infrastructure in town centres, including trees and greening, for cooling, shading, and aesthetic benefits. - Monitor heatwave and air pollution risks, particularly PM2.5, to identify vulnerable populations and reduce health impacts. - Recognise that heatwaves exacerbate pollution toxicity, integrating this into planning, monitoring, and public health strategies. |
| Health & wellbeing strategy | - Retrofit housing with insulation, ventilation, and energy efficiency measures to reduce indoor heat stress. - Identify and support heat-vulnerable residents using climate-health risk data. - Expand and improve green spaces, tree planting, and urban greening to provide shade, cooling, and better access to nature. - Incorporate building orientation, natural ventilation, and passive cooling in new and existing housing to reduce solar heat gain. - Promote access to cool open spaces for wellbeing and protection during heatwaves. - Ensure housing and urban planning support safe living in extreme heat and improve overall community resilience. |

## **S1.7. Housing interventions**

| **Strategy** | **Interventions** |
| --- | --- |
| Local plan | - Require high energy-efficiency standards for all new and existing homes to reduce fuel poverty, carbon emissions, and cold-related illness. - Promote retrofitting of existing housing, including historic and private rented homes, with insulation, heating upgrades, and energy-efficiency measures. - Deliver low-carbon or net-zero homes and low-carbon heat networks where feasible (e.g., district heating). - Encourage passive design and good building fabric (orientation, thermal mass, ventilation) to minimize energy demand. - Integrate flood-resilient construction for new and refurbished homes, including raised thresholds, safe escape routes, flood-proofing of utilities, and raised floor levels. - Locate new developments away from flood-prone areas, and implement Sustainable Drainage Systems (SuDS) and permeable surfaces to reduce surface runoff. - Use climate-adaptive design measures to mitigate overheating (ventilation, dual aspect layouts, green roofs, shading). - Require building materials and layouts that enhance resilience to extreme weather while reducing embodied carbon. - Ensure minimum space and amenity standards to reduce overcrowding and support wellbeing. - Require private outdoor space, communal areas, green infrastructure, tree planting, and access to parks or active travel routes to improve physical and mental health. - Sit housing away from major roads and pollution sources to protect respiratory and cardiovascular health. - Ensure adequate ventilation, damp mitigation, and thermal comfort to reduce mould and cold-related health risks. - Provide adaptable and accessible homes (e.g., M4(2)/M4(3) standards or equivalents) to support aging populations and residents with disabilities. - Promote flexible building designs to accommodate changing household needs or future climate adaptation. - Expand extra-care and supported housing for vulnerable residents to maintain independence and resilience. - Deliver large-scale housing with regeneration schemes integrating environmental, social, and climate-conscious design. - Expand supply of affordable and council homes, ensuring safety, warmth, and sustainability. - Improve quality in the private rented sector through licensing, minimum standards, and enforcement. - Bring empty or substandard properties back into use to reduce neighborhood decay and environmental hazards. - Address homelessness and improve temporary accommodation to reduce exposure to cold, damp, and overcrowding. - Apply sustainable design and construction standards (BREEAM Excellent/Very Good, energy- and water-efficient systems, sustainable materials). - Integrate green roofs, urban greening, and natural spaces within residential developments. - Support active travel infrastructure, cycle storage, and low-car development in housing schemes. - Promote site-specific masterplans that integrate green infrastructure, shading, stormwater management, and low-flood-risk layout. |
| Climate or Environment Strategy | - Retrofit existing homes to improve insulation, ventilation, airtightness, and indoor environmental quality, reducing cold, damp, and overheating risks. - Replace inefficient gas boilers with low-carbon alternatives, such as heat pumps, solar thermal, or connection to district heat networks. - Support large-scale retrofit programmes for council housing, housing associations, and private rented stock, prioritising homes with poor EPC ratings. - Provide financial support, grants, loans, or advice to homeowners and tenants to enable energy-efficiency upgrades and low-carbon heating installation. - Implement fabric-first or whole-building approaches to improve thermal efficiency and reduce heat loss. - Integrate overheating risk management and climate-adaptive measures into both retrofits and new developments, including shading, ventilation, orientation, and passive cooling. - Maximise renewable energy generation at home (e.g., rooftop solar PV, solar thermal) to improve energy resilience and reduce bills. - Improve flood resilience and climate adaptation in buildings, including SuDS, green roofs, water harvesting, and design for extreme weather. - Ensure new developments are low-carbon, energy-efficient, and climate-resilient, meeting high standards such as BREEAM Excellent or zero-carbon housing benchmarks. - Conduct targeted retrofits for hard-to-treat or heritage properties using specialist approaches and standards (e.g., Passivhaus Energyphit, PAS 2035). - Establish databases and mapping tools to identify vulnerable households and high-risk areas, supporting prioritised interventions for fuel poverty and climate risk. - Integrate retrofit, energy efficiency, and renewable energy programmes with wider urban planning, including collective retrofits, public building upgrades, and spatial frameworks for flood and heat risk. - Engage and upskill local installers, assessors, and supply chains to support effective retrofit delivery. - Promote monitoring, enforcement, and compliance with energy efficiency standards in private rented and commercial sectors. - Support residents in accessing retrofit schemes and grants through consumer-facing portals, advice services, and local authority programmes. - Consider the embodied energy and sustainability of building materials in retrofit and new build projects, aiming to minimise overall carbon footprint. - Deliver deep retrofit programmes targeting energy efficiency, low-carbon heat, and reduced overheating to tackle fuel poverty and improve health outcomes. |
| Net Zero or low carbon strategy | - Conduct home energy audits to identify opportunities for efficiency improvements. - Retrofit homes with insulation (walls, roofs, floors), double/triple glazing, and airtightness measures to reduce heat loss and cold-related illness. - Install energy-efficient lighting (e.g., LEDs) and improve overall building fabric. - Deploy low-carbon and renewable heating systems, including heat pumps, hydrogen-ready systems, solar thermal, and district heating networks. - Implement large-scale retrofit programmes in both social and private housing, including legacy poor-quality homes, targeting EPC improvements to at least C. - Support low-income households with grants, local schemes, and targeted interventions to reduce fuel poverty and ensure homes are warm in winter. - Provide guidance, advice, and financial support to property owners for sustainable retrofitting and energy-efficient upgrades. - Apply whole-building approaches to deep retrofit and new builds, integrating energy, water, and material efficiency in line with climate-conscious design standards. - Ensure new developments meet high sustainability and low-carbon standards, including BREEAM Excellent/Very Good ratings, and incorporate water conservation measures such as greywater recycling and rainwater harvesting where cost-effective. - Use planning and building regulations to set minimum energy efficiency requirements, encouraging retrofits and new builds that exceed standard regulatory levels. |
| Housing | - Retrofit existing homes to improve insulation, ventilation, airtightness, and thermal efficiency (fabric-first design), reducing cold, damp, mould, and overheating risks. - Upgrade heating systems with low-carbon alternatives (heat pumps, district heat, solar thermal) and phase out inefficient gas boilers. - Conduct large-scale retrofit programmes for council, housing association, and private rented stock, targeting EPC improvements (e.g., EPC C by 2025/2030). - Address poor-quality legacy housing (including tower blocks and historic properties) through refurbishment rather than demolition where feasible. - Install environmental sensors, smart meters, and temperature/humidity monitoring to support proactive energy and health interventions. - Implement fire safety retrofits, including sprinklers, fire door replacement, and removal of unsafe cladding in high-rise blocks. - Integrate flood resilience and surface water management in estates and new developments (SuDS, raised electrics, green roofs, reflective walls). - Improve indoor environmental quality: control humidity, reduce noise and pollution, and maintain safe air quality standards. - Ensure new builds meet zero-carbon, high sustainability, and Future Homes Standard requirements, including triple glazing, low-carbon heating, and reduced heat loss. - Apply space, accessibility, and adaptable housing standards (M4(2)/M4(3)) to support older adults, disabled residents, and climate resilience. - Conduct home condition surveys, stock appraisals, and Private Sector Stock Condition Surveys to identify hazards, disrepair, and climate-related risks. - Target interventions to fuel-poor and vulnerable households via grants, loans, WinterWatch schemes, and Warm Homes programmes. - Bring empty homes back into use through lease-and-repair schemes, empty homes loans, and advice/support services. - Support sustainable private rented sector housing through property and tenant accreditation schemes, rent deposit guarantees, and enforcement of hazards and energy-efficiency standards. - Address overcrowding through targeted allocation, new supply, and space standard enforcement to reduce health and heat exposure risks. - Apply water efficiency measures and energy/water-saving devices to reduce consumption in both new and retrofitted homes. - Support young people leaving care, older adults, and other vulnerable groups in accessing safe, energy-efficient, and climate-resilient housing. - Encourage modular, energy-efficient construction and whole-building retrofit approaches, including deep retrofit for hard-to-treat or heritage properties. - Prioritise neighbourhood-level interventions using data and intelligence platforms to target worst-performing areas and residents most at risk. - Promote community resilience through green infrastructure, cycle storage, active travel, and sustainable estate-wide upgrades. |
| Food & food waste | - Promote community food growing spaces on publicly owned land, housing estates, and under-used sites. - Integrate food-growing areas into new housing developments and landscaping schemes. - Encourage edible and pollinator-friendly planting in communal green spaces to support biodiversity and community wellbeing. - Enable Registered Providers to make non-productive land available for community food projects. - Support access to allotments and community gardens, ensuring equitable opportunities for older adults, vulnerable populations, and local residents. - Implement planning policies to prevent food deserts and food swamps by promoting healthy food access and engaging with local retailers. - Link food-growing and greening initiatives to broader outcomes such as flood resilience, social cohesion, and climate-adaptive urban design. - Promote the “Right to Grow” and similar initiatives to maximise the use of green spaces for community food production. |
| Flooding & coastal change | - Promote property-level flood resilience (PFR) for homes in high-risk areas, including flood gates, raised sockets/electrics, non-return valves, rapid-drying materials, and barriers. - Integrate Sustainable Drainage Systems (SuDS) in new housing developments, including permeable paving, rain gardens, tree pits, swales, retention ponds, and water butts/rainwater harvesting. - Retrofit SuDS and flood-mitigation measures on existing estates to improve surface water management. - Use planning policies, Strategic Flood Risk Assessments (SFRA), and the National Planning Policy Framework (NPPF) to avoid siting vulnerable housing in Flood Zones 2–3 unless essential. - Ensure new development meets greenfield runoff rates and does not increase flood risk elsewhere. - Maintain and restore floodplains, drainage systems, and riparian responsibilities to protect residential areas. - Encourage household and community action, including rainwater management and property-level flood measures, supported by community flood wardens where appropriate. - Target highest-risk and most deprived neighbourhoods first for flood resilience interventions. - Require climate change assessments in planning applications to ensure long-term resilience of housing. - Control inappropriate basement development and ensure drainage improvements in redevelopment sites. - Protect critical infrastructure (electricity, water, care facilities) through flood-aware planning and property-level measures. - Use local flood investigations (e.g., Section 19 reports) to inform future interventions and neighbourhood-level resilience planning. |
| Transport (active travel) | - Improve walking, cycling, and wheeling access to, from, and through housing estates and new developments, connecting homes to services, schools, and public transport. - Deliver infrastructure to support cycling, including secure cycle parking, bike hangars, segregated lanes, and safe crossings. - Design streets and public spaces with shade, shelter, and climate-resilient features to mitigate overheating and extreme weather. - Reduce traffic exposure in residential areas through Low Traffic Neighbourhoods, traffic filtering, lower speed limits, traffic calming, School Streets, and Play Streets. - Integrate private estate roads and local streets into public realm networks to ensure equitable, safe access for all users. - Require developers to embed active travel and low-carbon transport options in new developments, including EV charging, accessible pedestrian routes, and Travel Plans. - Ensure housing developments contribute to low-carbon, resilient town centres and reduce car dependency. - Reallocate road space and improve highway infrastructure to support safer walking and cycling, reduce congestion, and enhance resilience to climate pressures. - Enhance maintenance of streets and paths using smart monitoring (e.g., sensors for ice, potholes) to ensure safety and accessibility. |
| Greenspace or biodiversity strategy | - Increase tree canopy and shade provision in residential areas to improve cooling, reduce urban heat islands, and enhance air quality. - Integrate greenspace, parks, and green corridors into housing developments and regeneration areas to improve walkability, recreation, and access to nature. - Ensure local parks and informal greenspace are within standard walking distances (e.g., 400–600m) of homes, prioritising areas with deficiencies or high-density housing. - Use urban greening features such as green roofs, green walls, brown roofs, bird/bat boxes, and permeable surfaces to enhance biodiversity, reduce heat, and improve stormwater management. - Apply Biodiversity Net Gain and other planning standards to improve local green infrastructure and ecological connectivity. - Design parks and open spaces to be flood-resilient and integrate SuDS where possible to protect residential areas. - Restore rivers and watercourses to reduce downstream flood risk affecting housing. - Enhance maintenance, safety, lighting, and accessibility of green spaces to support physical activity, social cohesion, and mental health. - Provide financial contributions for play spaces and ensure equitable access to recreational areas in all new housing developments. - Reduce barriers to accessing green spaces, including severance from roads or private ownership, through underpasses, bridges, or negotiated access. - Encourage private garden greening and discourage full paving to enhance biodiversity, drainage, and cooling. |
| Air quality action plan | - Retrofit council and private sector homes to improve energy efficiency, insulation, and heating systems, targeting EPC improvements (e.g., EPC-B by 2030) and fuel-poor households. - Replace old boilers with low-emission or ultra-low NOx alternatives, including heat pumps, district heating, and other renewable systems (PV, solar thermal), and phase out gas and solid-fuel heating where feasible. - Promote and facilitate national and local grants, schemes, and finance options (e.g., RE:NEW, RE:FIT, LAD, HUG, SHDF) to support retrofits, low-carbon heating, and renewable energy installation. - Discourage domestic wood and coal burning through Smoke Control Areas, CleanBurn campaigns, public education, and regulatory enforcement. - Install carbon monoxide monitors in homes with fuel-burning appliances and promote ventilation standards to maintain safe indoor air quality. - Incorporate air-quality mitigation in planning and development, including:   - IAQM construction dust guidance and risk-based mitigation during demolition/construction.   - Air quality planning guidance for new housing developments.   - Mitigation measures in Air Quality Management Areas (AQMAs).   - Travel planning, reduced car dependency, EV charging, and green infrastructure integration to improve local air quality. - Monitor cumulative air-quality impacts of multiple housing developments and coordinate interventions via centralised databases. - Integrate energy-efficient and low-emission standards into new build developments, including compliance with Building Regulations (F & J) and promotion of clean heating systems. - Encourage developers and local authorities to adopt sustainable transport measures, EV readiness, and 20mph/traffic-calmed streets to reduce pollution exposure. |
| Health & wellbeing strategy | - Deliver programmes such as the Damp Charter to prevent damp, mould, and cold-related illness. - Conduct housing audits to assess quality, overcrowding, affordability, population needs, and security. - Identify vulnerable tenants and households through policies such as the Vulnerable Tenants Policy, Housing Needs Vulnerable Residents Policy, and evidence on climate-health vulnerability. - Develop neighbourhood-based, multi-agency approaches linking housing services with social prescribers, health partners, and community care providers. - Prioritise housing retrofit and insulation for fuel-poor and climate-health-vulnerable households. - Provide financial support, grants, and energy-efficiency programmes (e.g., Warm Homes, Energy Champions) to reduce fuel poverty and cold-related illness. - Prevent homelessness and support people in insecure, low-quality, or overcrowded accommodation. - Ensure housing is suitable for ageing populations, sheltered and supported housing residents, and those with long-term health conditions. - Design homes to be warm in winter and cool in summer, with shading, natural ventilation, tree cover, and energy-efficient orientation. - Promote sustainable construction and low-emission materials while balancing energy efficiency with indoor air quality (ventilation and filtration). - Retrofitting existing housing to support healthy living, ageing well, and independence. - Ensure homes are safe, decent, and of the right number, type, quality, affordability, and tenure to meet local population needs. - Monitor health and housing outcomes, including stable housing for people in contact with health services, housing benefit recipients, and households at risk of homelessness. - Engage wider partners, such as GPs, primary care, community care services, and ambulance trusts, to increase awareness of fuel poverty, energy efficiency, and housing support programmes. |

| **Region** | **Local Authority** | **Sector** | **Title** | **Year** |
| --- | --- | --- | --- | --- |
| London | Lambeth | Air quality | Air Quality Action Plan 2023-25 | 2023 |
| London | Lambeth | Air quality | Air Quality Action Plan 2017-2022 | 2017 |
| London | Lambeth | Air quality | JSNA Air Quality | 2019 |
| London | Barnet | Air quality | Air quality action plan 2023 - 2028 | 2023 |
| London | Newham | Air quality | Air Quality Action Plan 2019 – 2024 | 2019 |
| Manchester | Greater Manchester | Air quality | Greater Manchester Air Quality Action Plan 2016 - 2021 | 2016 |
| NE England | Hull | Air quality | Kingston upon Hull City Council 2017 Air Quality Strategy | 2019 |
| NE England | Northumberland | Air quality | Air quality strategy 2025 - 30 | 2025 |
| NE England | Newcastle Upon Tyne | Air quality | City of Newcastle upon Tyne Air Quality Action Plan | 2006 |
| SE England | West Sussex | Air quality | Breathing Better: Air quality plan | 2023 |
| SE England | Reading | Air quality | Reading Borough Council Air Quality Action Plan In fulfilment of Part IV of the Environment Act 1995 Local Air Quality Management 2024 - 2029 | 2024 |
| London | Lambeth | Environment & climate | Lambeth's Climate Action Plan | 2022 |
| London | Barnet | Environment & climate | Sustainability Strategy | 2022 |
| London | Newham | Environment & climate | Climate Emergency Action Plan | 2020 |
| London | Newham | Environment & climate | Newham Just Transition Plan | 2023 |
| Manchester | Greater Manchester | Environment & climate | 5-Year Environment Plan for Greater Manchester 2019-2024 | 2019 |
| Manchester | Greater Manchester | Environment & climate | 5-Year Environment Plan for Greater Manchester 2025-2030 | 2024 |
| Manchester | Greater Manchester | Environment & climate | Greater Manchester Resilience Strategy 2020-2030 | 2020 |
| Manchester | Tameside | Environment & climate | Climate Change & Environment Strategy 2021-2026 | 2021 |
| NE England | Northumberland | Environment & climate | Climate Change Action Plan 2021-23 | 2021 |
| NE England | Northumberland | Environment & climate | Climate Change Action Plan 2024-26 | 2024 |
| SE England | West Sussex | Environment & climate | Climate Change Strategy 2020 - 2030 | 2020-2030 |
| SE England | West Sussex | Environment & climate | Climate action and adaptation plan | 2024 |
| SE England | Kent | Environment & climate | Kent Environment Plan | 2025 |
| SE England | Reading | Environment & climate | The Reading Climate Emergency Strategy 2020-25 | 2020 |
| London | Lambeth | Flooding & coastal change | Lambeth Local Flood Risk Management Strategy 2014 - 2020 | 2014 |
| London | Barnet | Flooding & coastal change | Local Flood Risk Management Strategy | 2023 |
| London | Newham | Flooding & coastal change | Local Flood risk Management Strategy | 2024 |
| London | Newham | Flooding & coastal change | London Borough of Newham Level 1 & 2 Strategic Flood Risk Assessment | 2017 |
| Manchester | Salford | Flooding & coastal change | Salford Local Flood Risk Management Strategy | 2015 |
| Manchester | Greater Manchester | Flooding & coastal change | Greater Manchester Strategic Flood Risk Management Framework | 2018 |
| Manchester | Tameside | Flooding & coastal change | Tameside Flood Risk Management Strategy | 2016 |
| NE England | Hull | Flooding & coastal change | Local Flood Risk Management Strategy 2022 - 2028 | 2022 |
| NE England | Northumberland | Flooding & coastal change | Local flood risk management strategy | 2015 |
| NE England | Newcastle upon Tyne | Flooding & coastal change | Newcastle City Council Local Flood Risk Management Plan | 2016 |
| SE England | West Sussex | Flooding & coastal change | Local Flood Risk Management Strategy | 2025 |
| SE England | West Sussex | Flooding & coastal change | West Sussex Local Flood Risk Management Strategy (2013 – 2018) | 2013 |
| SE England | Kent | Flooding & coastal change | Kent Local Flood Risk Management Strategy 2017-2023 | 2011 |
| SE England | Kent | Flooding & coastal change | Local Flood Risk Management Strategy 2024 to 2034 | 2024 |
| SE England | Reading | Flooding & coastal change | Reading Borough Council Local Flood Risk Management Strategy | 2015 |
| London | Lambeth | Food & food waste | Food Poverty and Insecurity Action Plan | 2021 |
| London | Lambeth | Food & food waste | Lambeth Food Poverty and Insecurity Action Plan 2021-2024 | 2021 |
| London | Barnet | Food & food waste | Barnet Food Plan | 2022 |
| London | Barnet | Food & food waste | Food Secure Barnet: Action Plan 2019-2021 | 2021 |
| London | Newham | Food & food waste | Young People and Food Security Strategy | 2021 |
| Manchester | Greater Manchester | Food & food waste | Manchester Food Strategy 2023-2028 | 2023 |
| Manchester | Greater Manchester | Food & food waste | The Manchester Food Strategy 2023-2028 | 2023 |
| Manchester | Greater Manchester | Food & food waste | Food Poverty Action Plan for Greater Manchester: 2019-2022 | 2019 |
| Manchester | Tameside | Food & food waste | Tameside Good Food Plan Strategic Framework for Action 2024-2028 | 2024 |
| NE England | Hull | Food & food waste | Hull Food Action Plan 2019-2021 | 2019 |
| NE England | Hull | Food & food waste | A Food Strategy for Hull 2024 - 2029 | 2024 |
| NE England | Northumberland | Food & food waste | Northumberland Food Strategy 2024-27 | 2024 |
| London | Lambeth | Greenspace & biodiversity | Lambeth's Parks and open spaces | 2020 |
| London | Barnet | Greenspace & biodiversity | Parks and open spaces strategy 2025 - 2035 | 2025 |
| London | Barnet | Greenspace & biodiversity | Parks and Open Spaces: Our Strategy for Barnet 2016-2026 | 2016 |
| London | Newham | Greenspace & biodiversity | OUR GREENSPACES AND WATER SPACES | 2021 |
| Manchester | Salford | Greenspace & biodiversity | Salford greenspace strategy and supplementary planning document | 2019 |
| Manchester | Greater Manchester | Greenspace & biodiversity | The Greater Manchester Local Nature Recovery Strategy | 2025 |
| NE England | Hull | Greenspace & biodiversity | Hull City Council Open Space Strategy | 2016 |
| NE England | Northumberland | Greenspace & biodiversity | Northumberland Biodiversity Action Plan | 2008 |
| NE England | Newcastle Upon Tyne | greenspace & biodiversity | Newcastle Green Infrastructure Strategy | 2022 |
| SE England | West Sussex | Greenspace & biodiversity | Pollinator Action Plan 2019-2022 | 2018 |
| SE England | Kent | Greenspace & biodiversity | Kent Nature Partnership Biodiversity Strategy 2020 to 2045 | 2019 |
| SE England | Kent | Greenspace & biodiversity | Kent County Parks Strategy 2023 - 2028 | 2023 |
| SE England | Reading | Greenspace & biodiversity | READING OPEN SPACES STRATEGY UPDATE NOTE 2018 January 2018 | 2018 |
| London | Lambeth | Health & wellbeing | Health and Wellbeing Strategy 2023-2028 | 2023 |
| London | Barnet | Health & wellbeing | Barnet Joint Health and Wellbeing Strategy 2021 - 2025 | 2021 |
| London | Newham | Health & wellbeing | 50 Steps to a Healthier Newham Health and Wellbeing Strategy 2024-2027 | 2024 |
| Manchester | Greater Manchester | Health & wellbeing | Greater Manchester Integrated Care Partnership Strategy - Improving health and care in Greater Manchester 2023-2028 | 2023 |
| Manchester | Greater Manchester | Health & wellbeing | Mental Health and Wellbeing Strategy 2024-2029 | 2024 |
| Manchester | Tameside | Health & wellbeing | Building Back Fairer, Stronger, Together. Tameside Joint Health & Wellbeing Strategy and Locality Plan 2023-2028 | 2025 |
| NE England | Hull | Health & wellbeing | Health and Wellbeing strategy 2022 | 2022 |
| NE England | Northumberland | Health & wellbeing | Northumberland Joint Health and Wellbeing Strategy 2018-2028 | 2018 |
| NE England | Newcastle Upon Tyne | Health & wellbeing | A healthy, happy, and prosperous Newcastle Health and Wellbeing Strategy 2023 -28 | 2023 |
| SE England | West Sussex | Health & wellbeing | Joint Local Health and Wellbeing Strategy 2025 to 2030 | 2025 |
| SE England | West Sussex | Health & wellbeing | Creating healthy and sustainable places A public health and sustainability framework for West Sussex | 2020 |
| SE England | West Sussex | Health & wellbeing | West Sussex Joint Health and Wellbeing Strategy 2019 - 2024 | 2019 |
| SE England | Kent | Health & wellbeing | Kent Joint Health and Wellbeing Strategy | 2021 |
| SE England | Kent | Health & wellbeing | Kent 'Sustainability' JSNA Chapter Summary Update | 2014 |
| SE England | Reading | Health & wellbeing | Berkshire West Health & Wellbeing Strategy 2021-2030 | 2021 |
| London | Lambeth | Housing | Housing Strategy 2024-2030 | 2024 |
| London | Barnet | Housing | The Housing Strategy | 2023 |
| London | Barnet | Housing | Barnet Housing Strategy 2019 – 2024 | 2019 |
| London | Newham | Housing | Homes for people: Newham's Housing Delivery Strategy | 2020 |
| Manchester | Salford | Housing | Good Homes in Good Places: Salford’s Good Homes Strategy 2025-2030 | 2025 |
| Manchester | Greater Manchester | Housing | retrofitGM Accelerating the Renovation of Greater Manchester’s Buildings | 2021 |
| Manchester | Greater Manchester | Housing | Greater Manchester Housing Strategy 2019-2024 | 2019 |
| Manchester | Tameside | Housing | Tameside Housing Strategy 2021-2026 | 2021 |
| NE England | Hull | Housing | Hull’s Housing Strategy 2023 – 2030 | 2023 |
| NE England | Northumberland | Housing | Northumberland Housing Strategy for Northumberland 2019 - 2022 | 2019 |
| NE England | Newcastle Upon Tyne | Housing | Newcastle upon Tyne Development and Allocations Plan 2015 - 2030 | 2020 |
| NE England | Newcastle Upon Tyne | Housing | Plan for Council Housing 2024-2027 | 2024 |
| SE England | Kent | Housing | Kent and Medway Housing Strategy 2025 - 2030 Better Homes: Growth, Wellbeing and Choice | 2025 |
| SE England | Reading | Housing | Housing Strategy for reading 2020-2025 | 2020 |
| London | Lambeth | Local | Lambeth Local Plan 2020–2035 | 2021 |
| London | Barnet | Local | The Barnet Plan 2021-2025 | 2021 |
| London | Barnet | Local | Our Plan for Barnet 2023 to 2026 | 2023 |
| London | Barnet | Local | Barnet Local Plan 2021 - 2036 | 2021 |
| London | Newham | Local | Local Plan | 2018 |
| Manchester | Salford | Local | Salford Local Plan | 2020 |
| Manchester | Salford | Local | This is our Salford Corporate Plan 2024 to 2028 | 2024 |
| Manchester | Greater Manchester | Local | Our Manchester Strategy 2025-2035 | 2025 |
| NE England | Hull | Local | Hull City Council Corporate Plan 2018-2022 | 2018 |
| NE England | Hull | Local | Hull Local Plan 2016 to 2032 | 2017 |
| NE England | Northumberland | Local | Northumberland Local Plan | 2022 |
| NE England | Newcastle Upon Tyne | Local | Newcastle Local Plan | 2015 |
| SE England | Kent | Local | Framing Kent's Future - Our Council Strategy 2022 - 2026 | 2022 |
| SE England | Reading | Local | Reading Borough Local plan | 2019 |
| London | Barnet | Net zero/mitigation | Barnet Zero Annual report 2025 | 2025 |
| NE England | Hull | Net zero/mitigation | Hull 2030 Carbon Neutral Strategy | 2020 |
| NE England | Newcastle Upon Tyne | Net zero/mitigation | Net Zero Newcastle - 2030 Action Plan | 2020 [with updated report monitoring the plan in 2020-2021] |
| SE England | West Sussex | Net zero/mitigation | Achieving Net Zero Carbon by 2030 West Sussex County Council’s Carbon Management Plan | 2021 |
| SE England | Kent | Net zero/mitigation | Kent and Medway Energy and Low Emissions Strategy: Implementation Plan 2020-2023 | 2017 |
| SE England | Reading | Net zero/mitigation | The carbon plan 2020 -2025 | 2019 |
| London | Lambeth | Transport (active travel) | Transport Strategy | 2019 |
| London | Barnet | Transport (active travel) | Barnet Transport Strategy 2025 - 2042 | 2025 |
| London | Newham | Transport (active travel) | Mayors Transport strategy 2018 | 2018 |
| London | Newham | Transport (active travel) | Cycling Strategy 2017/18 – 2024/25 | 2017 |
| Manchester | Salford | Transport (active travel) | Transport In Salford 2025 | 2013 |
| Manchester | Greater Manchester | Transport (active travel) | Greater Manchester Transport Strategy 2040 | 2017, updated 2021 |
| NE England | Hull | Transport (active travel) | LOCAL TRANSPORT PLAN 3 STRATEGIC REFRESH & DELIVERY PLAN 2020 - 2026 | 2020 |
| NE England | Northeast: The Northeast Combined Authority | Transport (active travel) | Northeast Transport Plan 2021 - 2035 | 2021 |
| NE England | Newcastle Upon Tyne | Transport (active travel) | Northeast Transport Plan 2021 - 2035 Moving to a green, healthy, dynamic and thriving Northeast | 2021 |
| NE England | Newcastle Upon Tyne | Transport (active travel) | Newcastle Movement Strategy | 2025 |
| SE England | West Sussex | Transport (active travel) | West Sussex Active Travel Strategy 2024-2036 | 2024 |
| SE England | Kent | Transport (active travel) | Active Travel Strategy | 2019 |
| SE England | Kent | Transport (active travel) | Local Transport Plan 5: Striking the Balance | 2024 |
| SE England | Reading | Transport (active travel) | Local Transport Plan 4 (2024-40) Reading Transport Strategy 2040 | 2024 |
